# Aberrant T-cell phenotypes in a cohort of patients with post-treatment Lyme disease

**DOI:** 10.3389/fimmu.2025.1607619

**Published:** 2025-07-09

**Authors:** Alexander A. Girgis, Raffaello Cimbro, Ting Yang, Alison W. Rebman, Thelio Sewell, Daniela Villegas de Flores, Aarti Vadalia, William H. Robinson, Andrea L. Cox, Erika Darrah, Mark J. Soloski, John Aucott

**Affiliations:** ^1^ Lyme Disease Research Center, Division of Rheumatology, Department of Medicine, Johns Hopkins University School of Medicine, Baltimore, MD, United States; ^2^ Department of Biomedical Engineering, Johns Hopkins University, Baltimore, MD, United States; ^3^ Division of Immunology and Rheumatology, Department of Medicine, School of Medicine, Stanford University, Palo Alto, CA, United States; ^4^ VA Palo Alto Healthcare System, Palo Alto, CA, United States; ^5^ Division of Infectious Diseases, Department of Medicine, Johns Hopkins University School of Medicine, Baltimore, MD, United States

**Keywords:** post-treatment Lyme disease (PTLD), flow cytometry, immune phenotyping, post-acute infection syndromes (PAIS), cytokine profiling, T cells

## Abstract

Post-treatment Lyme Disease (PTLD) is a poorly understood complication of *Borrelia burgdorferi* infection with significant patient morbidity. Characterized by fatigue, generalized myalgias, and cognitive impairment, PTLD symptomatology closely resembles long COVID and other post-acute infection syndromes. While prior studies suggest immune dysregulation as a factor in PTLD pathogenesis, the mechanisms underlying its heterogeneous presentation and severity remain unclear. To associate symptom burden with discrete immune phenotypes, we applied factor analysis to self-reported symptom data from 272 PTLD patients to generate patient subgroups. We then immunophenotyped peripheral blood cells of these individuals and 28 healthy controls through 19-parameter flow cytometry and cytokine profiling to associate PTLD status and the newly defined subgroups with specific immune states. Our PTLD cohort had fewer circulating CXCR5+ CD4+ naïve T cells relative to healthy controls (5.2% vs. 8.3%, Padj < 0.001). These cells were positively associated with musculoskeletal pain in PTLD participants, but not healthy controls. This and additional immunophenotypic alterations, including an increased prevalence of CXCR3+ CCR4- CCR6- CD8 T cells (43.1% vs. 25.7%, Padj < 0.01), permitted the creation of an elastic net classifier which identified PTLD with moderate efficacy (AUC 0.83). Measurement of cytokines did not reveal associations with PTLD and did not improve the performance of the model. While we could not identify immune features which distinguished all patient subgroups, we did observe a female-specific increase in central memory CD8 T cells restricted to one high-fatigue patient subgroup. Additionally, factor analysis revealed multiple associations between immune cell frequency and the severity of specific symptoms. Collectively, our findings add to growing evidence of immune dysfunction as a prominent feature of PTLD.

## Introduction

Lyme disease is an inflammatory disease initiated by infection with *Borrelia burgdorferi* following a bite from an infected tick (1, 2). Signs and symptoms of early Lyme disease include the erythema migrans skin rash (EM), which may be accompanied by symptoms such as fever, sweats, chills, malaise, fatigue, and achiness. In some untreated cases, this early phase can evolve into systemic disease with signs of disseminated neurologic, cardiac, and/or joint involvement. Later, untreated patients can also develop late-onset musculoskeletal or neurological signs and symptoms (1–3). It is well known that both acute infection and late-onset disease can largely be controlled by appropriate antibiotic therapy and most individuals return to their pre-infection baseline. However, following proper diagnosis and treatment, there is a subset of patients which display a range of symptoms that can persist for years (4–6). This patient group is very heterogeneous, often self-reporting a variety of musculoskeletal, neurological, cognitive, and other symptoms. A research case definition for this illness, termed Post-Treatment Lyme Disease (PTLD), has been operationalized to capture a significant subset of these patients (4, 7). This definition includes a clear diagnosis of Lyme disease as well as continuing symptoms and functional changes that extend beyond 6 months after appropriate antibiotic treatment. This definition of PTLD has allowed investigators to recruit well-defined patient cohorts with the goal of understanding the complexity of PLTD, uncovering its underlying biology, and developing new approaches to diagnosis and treatment.

The underlying mechanisms driving PTLD are not known. Multiple hypotheses have been proposed, including the presence of persistent *B. burgdorferi* antigens and/or viable bacteria, post-infection neural network alternations, infection-induced autoimmunity, or generalized inflammation/immune dysregulation (4–6, 8). While findings suggestive of immunologic abnormalities in PTLD have been reported (9–13), a lack of well-defined alterations in specific cell populations presents a substantial barrier to understanding and treating patients with PTLD. It is important to note that the symptoms associated with PTLD are heterogeneous, and distinct mechanisms may drive disease pathophysiology in different patients. Linking immune phenotypes to specific symptoms presents an important step forward in understanding this heterogeneity.

Biological sex is known to alter many aspects of the immune system (14). Several prior studies have identified sex-based differences in prevalence of various stages of Lyme disease infection (15–18), as well as an increased risk of PTLD among females (19, 20). This is consistent with findings that females are more likely to develop post-acute infectious syndromes overall (21–24). However, the sex-specific immune characteristics involved in PTLD, and their relationship to potential sex-differences in symptom presentation, are not well understood. Therefore, an additional area of interest is the exploration of sex-specific immune features in PTLD patients.

To identify immune features associated with PTLD, we first applied factor analysis to self-reported symptom scores collected through the Post-Lyme Questionnaire of Symptoms (PLQS) from well-defined patients with PTLD. We then performed complex cellular immunophenotyping using flow cytometry and measured serum cytokines, chemokines, and acute-phase markers from the peripheral blood of these patients and healthy controls. By analyzing symptom profiles and immunophenotype data in tandem, we sought to link symptom burden and presentation to distinguish immune features within PTLD and identify differences between our PTLD cohort and healthy controls.

## Methods

### Study participants

Participants were recruited from a referral-based clinic population according to criteria previously described (7, 25). Briefly, participants (i) required a medical record indicating prior diagnosis and treatment for definite or probable Lyme disease according to Centers for Disease Control and Prevention criteria (26); (ii) experienced functionally impairing symptoms following acute Lyme infection and at the time of enrollment, including fatigue, musculoskeletal pain, and/or cognitive dysfunction; and (iii) were excluded for specific comorbidities including depression, cancer, HIV, and autoimmune disorders. The Institutional Review Board of the Johns Hopkins University School of Medicine approved this study, and written informed consent was obtained from all study participants (IRB00035457). Controls without a history of Lyme disease were recruited from a primary care clinic or through online advertising. To be eligible, they were required to have a negative two-tier serologic test for antibodies to *B. burgdorferi*, and self-report no prior Lyme disease diagnosis. They were also excluded for the same list of comorbidities as the patients with PTLD. Self-reported gender was obtained for all participants. Additional self-report of sex assigned at birth was added later in data collection and was therefore incomplete. We have elected to use the term “sex” in this manuscript under the assumption that observed differences are most likely to result from biological determinants; however, it is ultimately challenging to discern the relevance of social factors in patient outcomes. Given how our data were collected, we cannot be certain that we have captured biological sex for a small proportion of participants.

Self-reported symptom data from 272 participants collected using the PLQS were used in clustering analysis to generate subgroups of patients based on symptom profiles. Serum cytokine/chemokine profiling was performed on 258 participants and 28 healthy controls. Flow cytometry was performed on whole blood collected during study visits from 144 participants and 20 healthy controls, of whom all but four patients with PTLD underwent cytokine/chemokine profiling. One hundred and thirty-seven patients with PTLD had PLQS responses, cytokine data, and flow cytometry data available. Cohort overlap between the three data modalities is shown in **Supplementary Figure S1**.

### Demographic comparisons

We compared our healthy control cohort to PTLD participants for differences in demographic characteristics as well as co-morbidities. Among PTLD patient subgroups, we additionally tested for differences in acute infection characteristics. Categorical variables were compared using a Chi-square test, or Fisher’s exact test when analyzing low frequency events (<=5 occurrences). Continuous variables were compared using the univariate testing procedure described below.

### PLQS cluster analysis

The PLQS is a 36-item questionnaire previously used to define patient-reported symptoms of PTLD (7). For each symptom, presence and severity were assessed on a 4-part Likert scale (“absent” [0], “mild” [1], “moderate” [2], “severe” [3]). Thirty of the symptom questions on the PLQS were transformed into six latent factors according to an exploratory analysis conducted previously (25). Consistent with this prior study, the remaining six PLQS symptoms, which were both lowly endorsed and of limited clinical relevance, were not considered for analysis. The six composite factors are as follows: Fatigue/Cognitive, Ocular Disequilibrium, Infection-Type, Mood-Related, Musculoskeletal Pain, and Neurologic. PCA and k-means clustering was performed on normalized factor score data to generate between 2 and 20 clusters of patients. The number of six subgroups was chosen as a balance between adequately resolving cohort heterogeneity, maximizing silhouette score (27),and maximizing additional cluster performance parameters ADM, AD, FM, and FOM reported through the R package *ClValid (* 28).

### Sample acquisition

Sera was isolated using SST tubes, aliquoted, frozen at -80°C and thawed prior to cytokine measurement. Whole blood was assayed using flow cytometry on the same day blood was drawn, conducted over a multi-year period 2014–2018 as described previously (29).

### Cytokine and chemokine measurement

The Bio-Plex bead array system using Luminex xMAP technology was employed to perform multiplex analysis of 34 cytokines, chemokines, and acute-phase markers (30). Data processing was completed using Bio-Plex manager software (version 5.0). The final list of cytokines, chemokines, and inflammatory markers included in the analysis is shown in **Supplementary Table S1**. The protocol and data generated were minimum information about a microarray experiment (MIAME) compliant (31).

### Flow cytometry acquisition and analysis

Freshly drawn whole blood was stained using a 19-parameter panel designed to measure T cell, B cell, Monocyte, and NK cell populations (**Supplementary Table S2**). In a 12x75mm cell culture tube, 200uL of whole blood with 0.0025% sodium azide was combined with the antibody cocktail and Live/Dead fixable blue stain (Molecular Probes) for 35 minutes. Erythrocytes were removed by treating samples with Pharm Lyse (BD) for 15 minutes. Remaining cells were fixed with PFA and stored in PBS at 4°C until data collection. Data was acquired on a five laser FACS Aria II (Becton Dickinson) using FACSDiva software on the same day blood was drawn.

Analysis of the flow cytometry data was performed using FCSExpress (DeNovo Software) to generate 119 cell populations. Lymphocyte gating leveraged a previously published strategy measuring seven lineage markers using two fluorochromes (29). Some populations were overlapping classifications of the same cells, for example, gating effector memory T cells on a CD57 and HLA-DR quadrant gate as well as separately gating on binary CXCR5 positivity. The gating tree is described in detail in **Supplementary Figure S2** and **Supplementary Data**. Statistical tests were performed on the frequency of cells within each population expressed as a percentage of the parent gate. In situations with binary gating (e.g. CXCR5 positive and CXCR5 negative cells), only one gate was considered for analysis.

### Univariate testing

Individual variables, whether flow cytometry gate percentages or cytokine levels, were compared between groups as follows. The normality of each variable was assessed via Shapiro-Wilk test with alpha=0.05. Variables which failed normality criteria were tested using either a Wilcoxon rank-sum test (in the case of two groups) or a Kruskal-Wallis test. Those which satisfied normality criteria were tested using a t-test (in the case of two groups) or one-way ANOVA. For two-group comparisons, a student’s t-test was used in the case of equal variance and a Welch’s t-test for unequal variance. False-discovery rate (FDR) correction was performed using a Benjamini-Hochberg correction with alpha=0.05 (32). Flow cytometry and cytokine data were considered separately for the purposes of FDR correction. All cytokine data were adjusted via Box-Cox transformation prior to univariate significance testing.

### Confounder adjustment

Significantly different variables between groups as outlined above were additionally included as dependent variables in a generalized linear model (GLM) to adjust for possible confounders (33). The following variables were incorporated as independent variables of a GLM in addition to the immune feature of interest: Age, sex, race, and income status. For flow cytometry data, the year of data collection was also included. Group identity was coded as the independent variable of interest. This was either PTLD case vs. healthy control status, or for comparisons among participants with PTLD, the PTLD patient subgroup. Immune features were considered significantly different between groups only if they yielded a statistically significant coefficient in the resultant GLM. Residuals of each GLM were checked for normality using the *simulateResiduals* function in R package *DHARMa (* 34). GLMs with non-normal residuals were discarded and re-made following Box-Cox transformation of the immune feature of interest.

### Symptom associations with immune variables

We measured correlations between immune variables and each of six PLQS factor scores (Fatigue/Cognitive, Ocular Disequilibrium etc.). Spearman’s rho correlation coefficient was computed, and the FDR was controlled using the Benjamini-Hochberg procedure with alpha=0.05 for multiple testing correction (32). Significant correlations were further verified by generating a GLM with the immune feature as the dependent variable, and factor score as an independent variable along with participant age, sex, and race. For flow cytometry data, the year of sample collection was also considered. Residuals of each GLM were checked for normality using the *simulateResiduals* function in R package *DHARMa (* 34). GLMs with non-normal residuals were discarded and re-made following Box-Cox transformation of the factor score of interest.

### Elastic net regression

One-hundred and forty PTLD participants and 20 healthy controls with both flow cytometry and cytokine data were used to construct and test an elastic net classifier. Seventy percent of the cohort was used to train the model and 30% withheld as a test cohort. Training and test datasets were stratified to maintain equal sex and PTLD/healthy proportions. A 3-fold cross-validation elastic net regression was calculated using the function cv.*glmnet* from package *glmnet* in R (33). The model was constructed 100 times, recording coefficients, AUC, and misclassification error for each iteration. Of the 100 iterations of the model, the regression matching median AUC was selected as the representative model.

## Results

### Symptom profiling of PTLD participants

Cluster analysis was performed on self-reported PLQS symptoms from 272 participants. Consistent with prior reports, this cohort has a broad age range (median 48 years, 37–58 IQR), similar sex representation (56.6% male), and marked white, non-Hispanic skewness (91.5%) (**Table 1**) (7, 25). Consistent with the methods of a prior published study, the results of 30 questions on the PLQS were transformed into six factor scores representing separate manifestations of symptoms: Fatigue/Cognitive, Ocular Disequilibrium, Infection-Type, Mood-Related, Musculoskeletal Pain, and Neurologic (25). Each factor score was normalized to a mean of zero and standard deviation of one. We then performed K-means clustering on a matrix of normalized factor scores to generate six patient subgroups with distinct symptom profiles.

**Table 1:** Characteristics of 285 PTLD patients and 28 healthy controls with PLQS data, flow cytometry, and/or cytokine/chemokine data available. Extended demographic information, including by patient subgroup, is available in **Supplementary Data**.

Variable	PTLD Mean	PTLD Median	PTLD IQR	PTLD %Missing	HC Mean	HC Median	HC IQR	HC %Missing
Age (yrs)	48.26	49	22	.	42.29	37	26.75	.
Male (%)	56.5	.	.	.	39.3	.	.	.
White, non-Hispanic (%)	91.2	.	.	.	64.3	.	.	.
BMI	27.0	25.82	6.89	7.38	27.57	25.78	5.78	75
High-Income (> $100k/yr) (%)	56.1	.	.	.	32.1	.	.	.
STTT Seropositive (%)	85.1	.	.	1.05	.	.	.	.
EM Rash (%)	39.6	.	.	.	.	.	.	.
Time post-infection at enrollment (years)	3.20	1.73	3.45	.	.	.	.	.
Time from inf. to treatment (days)	208.9	22	123	.	.	.	.	.
Total time on antibiotics (days)	102.85	51	69.25	.	.	.	.	.
Lyme arthritis (initial inf.) (%)	10.66	.	.	.	.	.	.	.
Lyme carditis (initial inf.) (%)	1.47	.	.	.	.	.	.	.
Neuroborreliosis (initial inf.) (%)	6.62	.	.	.	.	.	.	.

**Figure 1:**
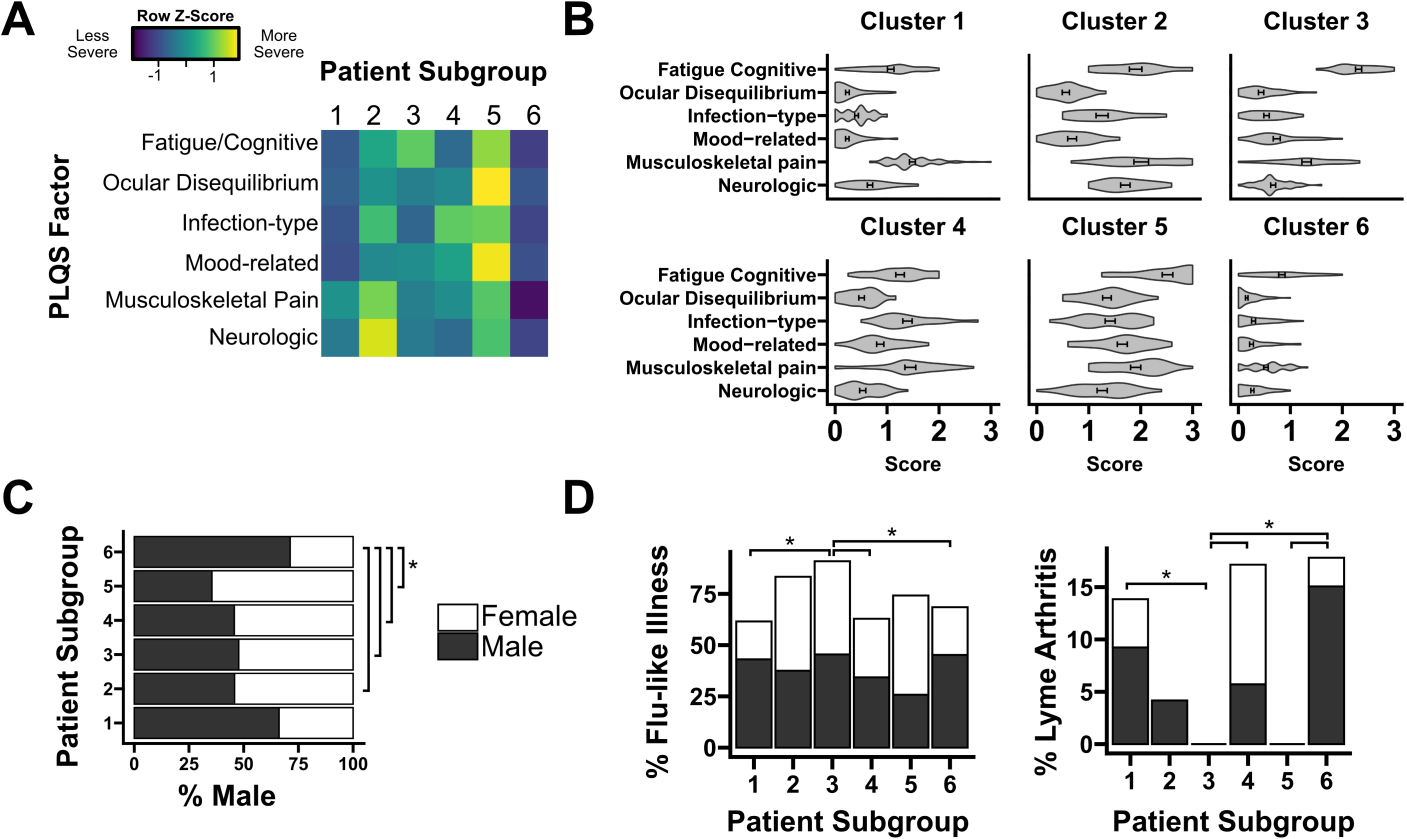
Cluster analysis of symptom profiles in PTLD. **(A)** Row-normalized factor scores for each of six PTLD patient subgroups. Color denotes the relative endorsement of each PLQS symptom factor. **(B)** Symptom profile for each patient subgroup, displayed on an absolute scale (“absent” [0], “mild” [1], “moderate” [2], “severe” [3]). **(C)** Sex composition of each patient subgroup. Group 6, with a comparatively mild symptom profile, is significantly more male than subgroups 2-5. **(D)** Two characteristics of primary Lyme infection, the prevalence of flu-like illness and the development of Lyme arthritis, are significantly different between patient subgroups. Bars are shaded by sex composition similarly to **(C)**. * = Padj < 0.05.

Symptom profiles generated for each cluster were broadly consistent with prior studies, with patient stratification by both overall intensity of symptoms as well as separation driven by specific factors. Cluster 5 participants highly endorsed all six symptom factors, while Cluster 6 participants experienced comparatively mild symptoms in every category (Mean factor score: 1.7 vs. 0.4, p<0.001). Cluster 2 had an especially high endorsement of the “Neurologic” factor, while Cluster 3 was dominated by the “Fatigue/Cognitive” factor (**Figure 1A**). For five of six clusters, the “Fatigue/Cognitive” factor was most highly endorsed, with the exception of Cluster 1, which more highly endorsed the factor “Musculoskeletal Pain” (**Figure 1B**). The heterogeneity of disease manifestations is consistent both with prior studies of PTLD as well as related work in ME/CFS, Long Covid, and other post-acute infection syndromes (22, 25, 35). We next sought to identify differences in patient characteristics among PLQS patient subgroups. We tested for differences in demographic characteristics including age, sex, and race as well as a variety of acute and post-acute infection characteristics including the presence of an EM rash or the development of complications such as Lyme arthritis, carditis, or neuroborreliosis. Prominent sex bias was observed in “mild symptom” cluster 6, which was significantly more male than “severe symptom” cluster 5 (71% vs. 35% male, Padj < 0.05, **Figure 1C**). This cluster also had a greater incidence of initial Lyme arthritis (17%) than female-enriched clusters 3 and 5, which had no Lyme arthritis (Padj <0.05, **Figure 1D**). Lastly, “Fatigue/Cognitive” cluster 3 was found to have a greater incidence of flu-like illness symptoms during acute infection than several other clusters (Padj < 0.05). Consistent with these findings, male patients have previously been observed both to have reduced symptom severity in PTLD relative to females, and a greater incidence of Lyme arthritis following acute infection (4, 7, 23). Broadly, our symptom profiles reinforce and refine existing knowledge of symptom heterogeneity in PTLD. With these patient subgroups in mind, we sought to conduct immune profiling of PTLD participants and associate objective immune findings with PTLD status and self-reported symptom profiles.

### Luminex cytokine profiling

**Figure 2:**
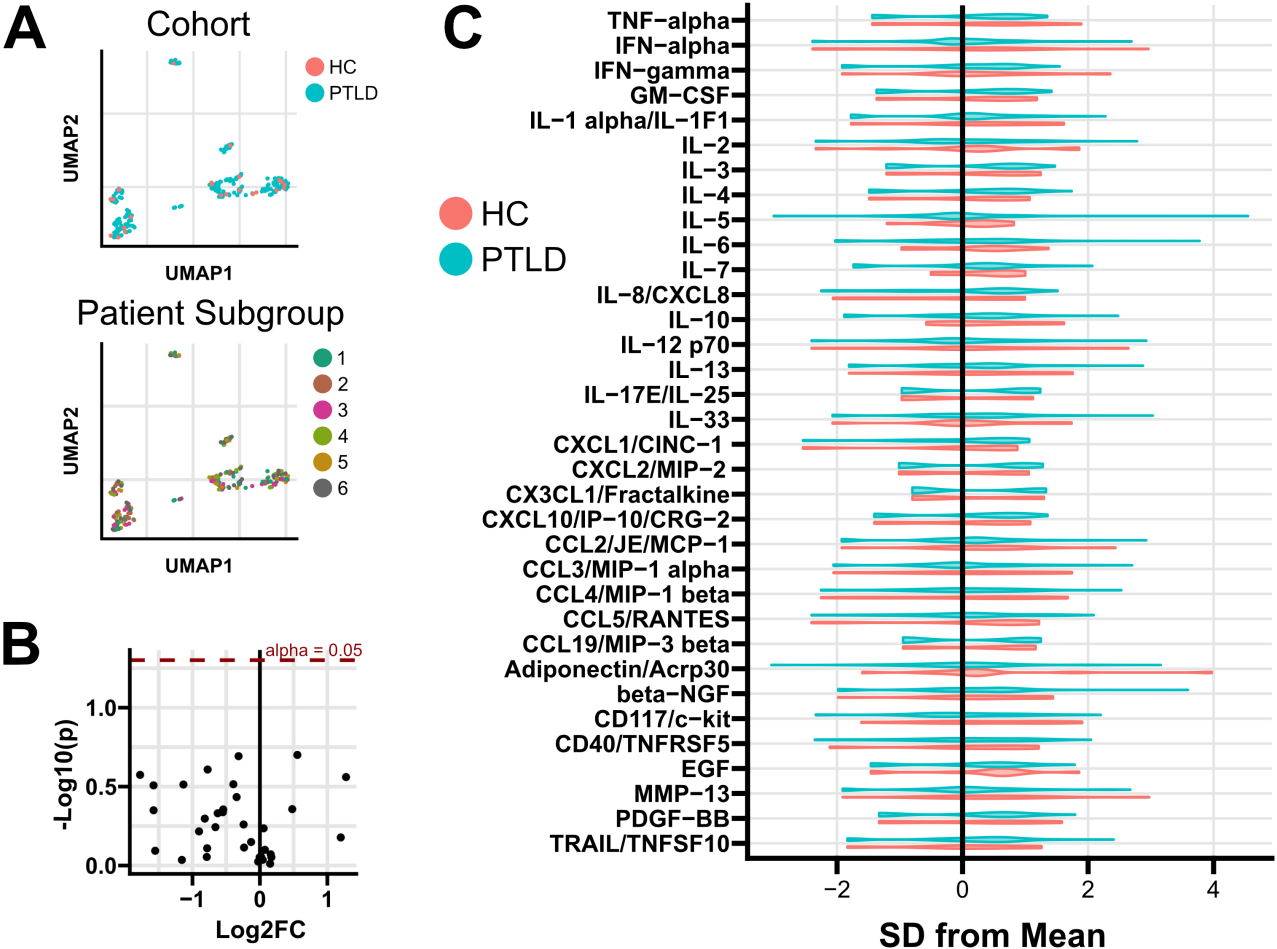
Cytokine and chemokine profiling of PTLD and healthy controls. **(A)** While heterogeneity in cytokine profiles was observed, multivariate clustering failed to separate subjects based on either PTLD/control status or by PTLD patient subgroup. **(B)** Univariate testing failed to identify differences between PTLD subjects and healthy controls. Positive Log2FC indicates overexpression in PTLD; unadjusted p values are shown. **(C)** Normalized distribution of cytokine expression in PTLD and healthy control cohorts. Additional testing failed to identify cytokines which varied among PTLD patient subgroups, or associate cytokine expression with PLQS symptom scores (**Supplementary Files S4, S5**).

Sera from 258 PTLD participants and 28 healthy controls were tested for 34 cytokines and chemokines using the Luminex platform (**Supplementary Table S1**). A multi-variate analysis was performed using dimensional reduction and k-means clustering. Heterogeneity in cytokine profile was noted across participants, with distinct patterns of cytokine expression (**Supplementary Figure S2**), but this heterogeneity did not associate with either PTLD status or PTLD symptom subgroup (**Figure 2A**). Univariate testing was performed comparing serum concentration of each analyte across patient subgroups. No significant differences by patient subgroup were identified. Similarly, no differences were observed in any analyte between the PTLD cohort as a whole and healthy controls (**Figures 2B, C**). No individual cytokines were found to significantly correlate with symptom endorsement for any of the six PLQS factor scores. Due to the importance of sex on both the prevalence and symptomatology of PTLD, we repeated all analyses on sex-stratified cohorts (**Supplementary Files S4, S5**). Sex-specific testing did not identify any differences between HC and PTLD cohorts or identify differences in cytokine profiles across patient subgroups.

### Flow cytometry profiling

**Figure 3:**
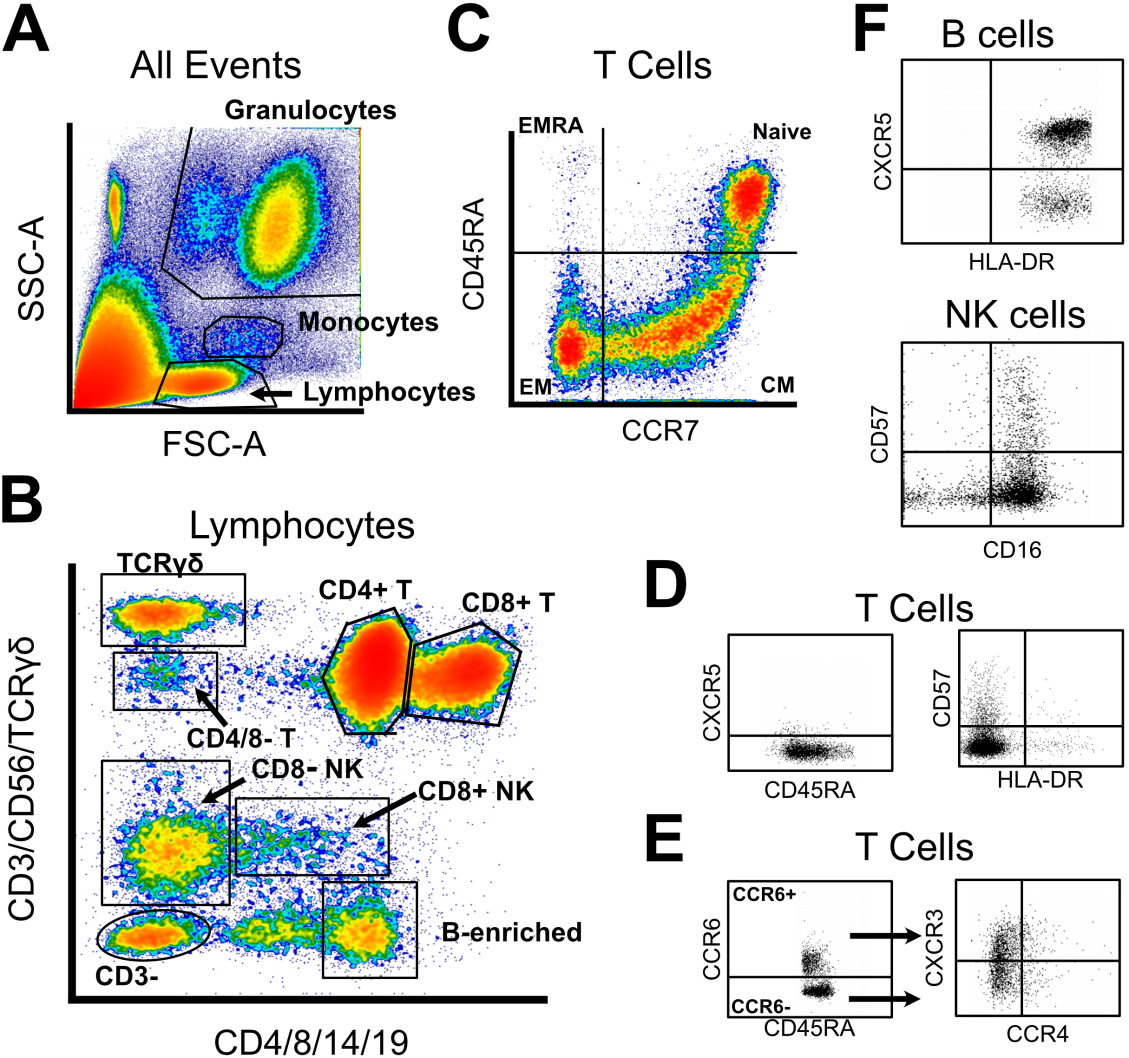
Abbreviated gating strategy for flow cytometry profiling. **(A)** Forward and side scatter were used to broadly gate lymphocytes, monocytes, and granulocytes. **(B)** Lymphocyte lineages were identified via a two-fluorochrome strategy, published previously. **(C)** CD4 and CD8 T cells were gated into naïve and memory subsets via CCR7 and CD45RA. **(D)** All T cell subsets were assayed for CXCR5, CD57, and HLA-DR expression. **(E)** In parallel, all T cell subsets were classified into Th1/2/9/17 subtypes using expression of CCR6, CCR4, and CXCR3. **(F)** Limited phenotyping of B and NK cell subsets was performed using CXCR5/HLA-DR or CD57/CD16 expression, respectively. Unabridged gating strategy is available in **Supplementary Data**.

**Figure 4:**
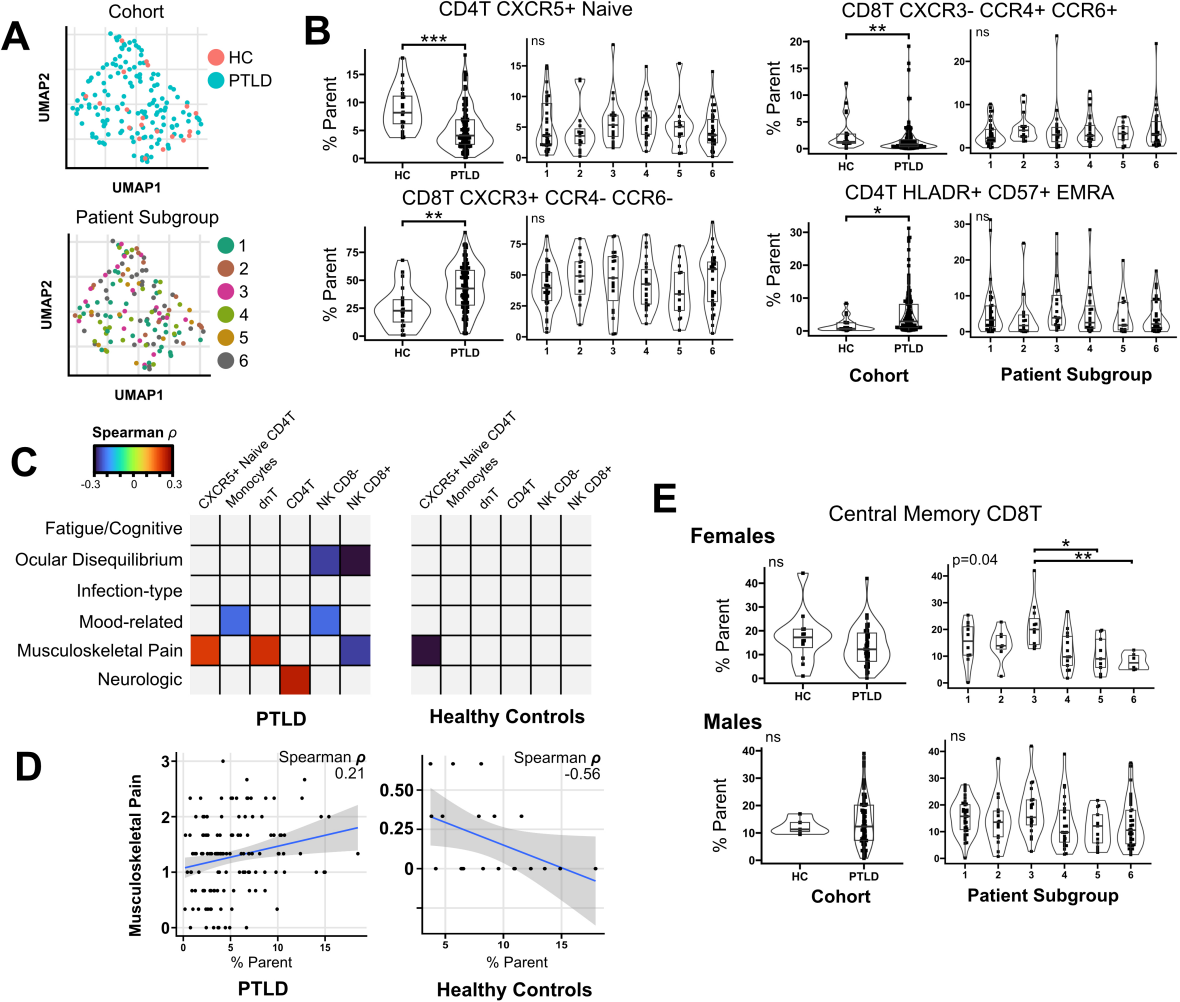
Flow cytometry profiling of PTLD subjects and healthy controls. **(A)** Multivariate clustering on flow cytometry features failed to differentiate PTLD from healthy controls, or separate individuals by patient subgroup. **(B)** Univariate testing identified four cell populations which differed between healthy controls and PTLD subjects, but these features did not vary based upon PTLD patient subgroup. **(C)** Heatmap of spearman correlation coefficients for correlations between immune features and PLQS symptom scores. Multiple cell populations correlated with PLQS factor scores (Padj < 0.05). Correlations which did not meet significance criteria are shaded grey. **(D)** Notably, CXCR5+ Naïve CD4 T cells had opposite correlations with musculoskeletal pain in PTLD and healthy control groups. **(E)** Testing by sex revealed female-specific variation in central memory CD8T cells across patient subgroups. For patient subgroup plots, multivariate (Kruskal-Wallis or Anova) P values displayed in upper left. For pairwise comparisons, * = Padj < 0.05; ** = Padj < 0.01, *** = Padj < 0.001.

Using a previously developed gating strategy, we phenotyped whole blood from 144 PTLD and 20 healthy control participants using a 19-parameter flow cytometry panel (**Figure 3**, **Supplementary Table S2**). Data was processed to generate a table of cell frequencies in each of the 119 manually derived gates. Cell counts within each gate, expressed as the frequency of the parent gate, were considered separate variables for statistical testing. Similarly to cytokine analysis, we sought to query whether the prevalence of specific cell populations distinguished PTLD symptom subgroups, or distinguished PTLD individuals from healthy controls. Multivariate dimensional reduction and k-means clustering did not generate clusters which stratified by PTLD status, or by PLQS patient subgroup (**Figure 4A**).

Univariate testing did not reveal differences between PTLD patient subgroups. However, multiple features varied between healthy controls and PTLD participants (**Table 2**). CXCR5+ Naive CD4 T cells were reduced in PTLD compared to healthy controls (8.8% vs. 5.2%, Padj. < 0.001). CD4+ effector memory CD45RA+ (EMRA) cells were increased in the PTLD cohort (5.1 vs. 1.6%, Padj < 0.01, **Figure 4B**). We also examined CXCR3, CCR4, and CCR6 on CD8 T cells and identified CD8+ Th1-like cells (CXCR3+ CCR6- CCR4-) which were markedly increased in PTLD relative to healthy controls (43.1% vs. 25.7%, Padj < 0.01), with a corresponding decrease in CD8+ Th1/17-like cells, defined as CXCR3- CCR6+ and CCR4+ (3.9% vs. 13.4%, Padj < 0.01). Lastly, CD4+ EMRA cells were increased in the PTLD cohort (5.1 vs. 1.6%, Padj < 0.01, **Figure 4B**).

**Table 2:** Flow cytometry populations which were significantly different between PTLD and healthy control cohorts, alpha < 0.05.

Population	% HC	% PTLD	Padj
CXCR5+ CD4 Naïve	8.84	5.17	0.001
CD8 Th1/17	13.43	3.88	0.003
CD8 Th1	25.68	43.15	0.003
CD57+ HLADR+ CD4 EMRA	1.58	5.12	0.024
CD57+ HLADR- CD8 Naive	1.86	3.09	0.073
CCR6- CD4 EMRA	54.91	68.37	0.081

Although no flow cytometry characteristics were significantly associated with PLQS symptom clusters, several parameters were correlated with individual PLQS factor scores (**Table 3**, **Figure 4C**). Most notably, CXCR5+ Naive CD4 T cells were positively associated with the “Musculoskeletal Pain” factor in the PTLD cohort (rho = 0.21, Padj= 0.041), but negatively associated with this factor in healthy control data (rho = -0.56, Padj = 0.017, **Figure 4D**). Multiple PLQS factors were also associated with NK cell frequencies.

**Table 3:** Significant correlations between flow cytometry populations and PLQS factor scores within the PTLD cohort, alpha < 0.05.

Population	PLQS Factor	Spearman ρ	Padj
NK CD8+	Ocular	-0.295	0.012
NK CD8+	MSK Pain	-0.254	0.026
NK CD8-	Ocular	-0.263	0.026
CD4T	Neurological	**0.246**	0.027
NK CD8-	Mood	-0.227	0.034
dnT	MSK Pain	**0.227**	0.034
Monocytes	Mood	-0.226	0.034
CXCR5+ CD4 Naïve	MSK Pain	**0.208**	0.041
NK CD8+	Fatigue/Cognitive	-0.213	0.047

Positive correlations are shown in bold.

Similarly to our treatment of cytokine data discussed previously, we repeated all analyses on sex-stratified cohorts. In comparing healthy controls to PTLD, changes in immune features tended towards the same direction in both sexes and do not appear biologically distinct (**Supplementary Figure S4**). In correlating PLQS factor scores with flow cytometry features, significant correlations observed in the combined sex cohort had the same direction of change in sex-stratified analyses, but did not meet significance criteria at alpha = 0.05. However, in associating flow cytometry data with PLQS patient subgroups, we observed sex-specific biases in central memory (CM) CD8 T cells. For females, patient subgroup 3 expressed a higher prevalence of CM CD8 T cells relative to other symptom subgroups (21.2% vs 12.2%, Padj = 0.04), which was not observed in male participants or when sexes were combined (**Figure 4E**).

### Multimodal elastic net classifier

**Figure 5:**
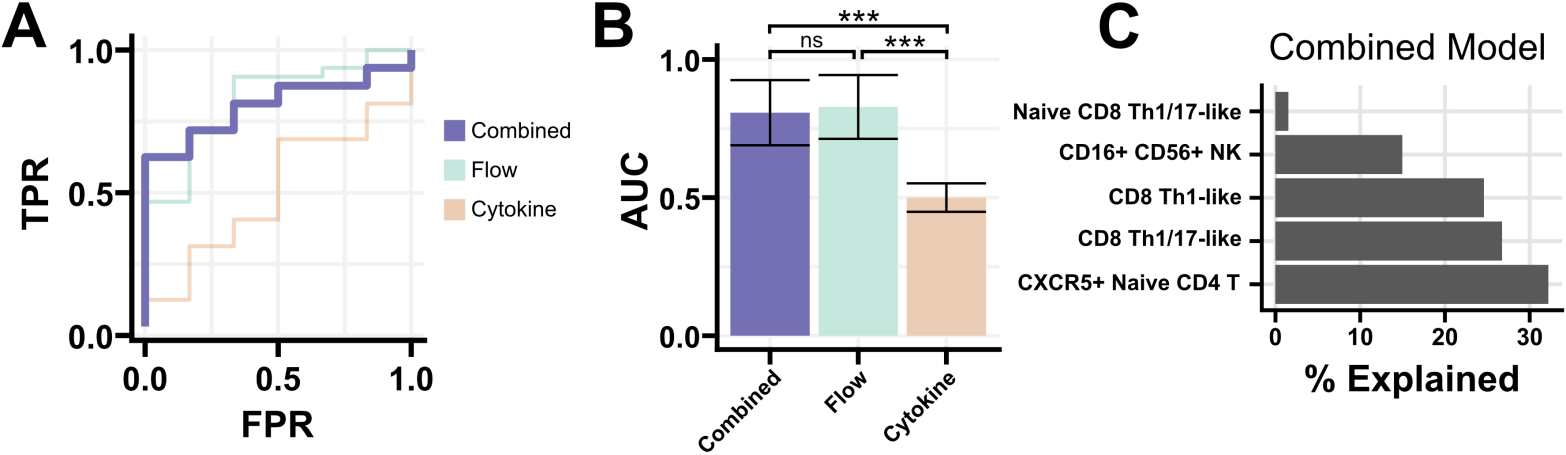
Elastic net regression for a multi-modal classifier. **(A)** Receiver-operating characteristic curve for models generated using flow cytometry and cytokine data, or either modality individually. The multi-modal classifier was moderately successful at identifying PTLD from healthy controls (AUC 0.83, misclassification error 0.26). **(B)** AUC of 100 iterations of the classifier using either all data, flow cytometry data alone, or cytokine data alone. The inclusion of cytokine data provided no improvement in performance over flow cytometry data alone. Mean +/- standard error shown. **(C)** Coefficients of a representative multi-modal classifier, expressed as the proportion each coefficient contributes to the model. Flow cytometry populations were solely responsible for classifier performance, with no contribution from cytokine data. *** = Padj < 0.001.

We next sought to combine cytokine and flow cytometry features for participants with both data collected. Despite the lack of statistically significant findings in cytokine data, we hypothesized that a combination model with flow cytometry features might reveal immune signatures associated with PTLD or its symptom manifestations. One-hundred and forty of the 144 PTLD participants with flow cytometry data, along with all 20 healthy controls, had cytokine data available. We combined cytokine and flow data and used a 3-fold cross validation elastic net classifier to separate PTLD participants from healthy controls. Classifiers based solely on flow cytometry data had a similar performance to the multimodal model, but classifiers trained on cytokine data were ineffective (**Figures 5A, B**). In a representative multimodal model, CXCR5+ Naive CD4 T cells were the most heavily weighted feature with 32% of variance explained. CD8 Th1-like and CD8 Th1/17-like cells were the next most heavily weighted coefficients, explaining approximately 25% variance each (**Figure 5C**). No cytokines were assigned predictive weight in any of the 100 iterations of the model. We attempted a similar approach to generate regressions which might predict PLQS factor scores for PTLD participants but were unsuccessful. Models were unable to predict any of the six PLQS factor scores with Spearman’s Rho > 0.12 and failed to produce non-zero coefficients in 49% of iterations (**Supplementary Table S3**).

## Discussion

Similarly to other post-acute infection syndromes, individuals with PTLD experience a wide variety of symptoms, which may be the result of distinct disease processes in different patients. Therefore, successfully identifying immune correlates of disease may require deconstructing patient populations into subgroups based on symptom presentation. Using a factor analysis previously employed in this cohort, we generated subgroups of PTLD based on self-reported symptoms on the PLQS. Following the creation of patient subgroups, we queried whether immune features might distinguish specific subgroups or collectively differentiate our PTLD cohort from healthy controls. Our factor analysis of self-reported symptom data from the PLQS demonstrated heterogeneity in the symptom burden of PTLD patients consistent with prior reports (7, 25). Similar to those studies, we note heterogeneity in symptoms and symptom profiles which are enriched for male or female participants. We deliberately increased the clustering resolution of our analysis relative to prior work to identify patient subgroups with increased granularity. Our findings provide a framework to associate subjective symptom reporting with objective immune phenotypes.

We were unable to identify differences between healthy controls and PTLD participants in the levels of serum cytokines or chemokines, nor were we able to associate soluble immune mediators with symptom burden. Furthermore, these data were not predictive of PTLD when used in a multi-modal predictive model, which relied wholly upon flow cytometry data to distinguish PTLD from healthy controls. Prior analyses in longitudinal cohorts of Lyme disease patients followed from the time of acute infection have noted trends in CCL19, IL-23, and IFNα expression associated with the development of PTLD (9–12). Several possibilities may explain this discrepancy with our findings. First, participants in this analysis are from a retrospective cohort of PTLD individuals with enrollment substantially later than initial infection (median enrollment 635 days post-infection, IQR 222 – 1484). Prior studies have not analyzed participants more than 1-year post-infection. Additionally, variability in enrollment criteria across different cohorts with regards to the presence of EM rash, seropositivity, cohort catchment area, and other factors complicates direct comparison across cohorts. Rather than contradicting prior findings, these studies may describe temporally and clinically distinct PTLD populations following Lyme infection.

Though our study was primarily powered to explore clinical heterogeneity within the PTLD population, we identified multiple aberrations in T cell populations that distinguish individuals with PTLD from healthy controls. PTLD patients had decreased frequencies of CXCR5+ CD4 naïve T cells, an increased prevalence of CD8 Th1-like cells (with a corresponding decrease in Th1/17-like cells), and an increased prevalence of CD57+ HLA-DR+ CD4 EMRA cells.

CXCR5 is the chemokine receptor for CXCL13, a chemokine responsible for lymphocyte recruitment to lymphoid follicles and a defining marker of follicular-helper T cells (T_FH_) (36, 37). T_FH_ cells are critical mediators of T cell help in humoral immunity, facilitating B cell maturation and robust antibody responses (36). The CXCR5+ naïve CD4 T cells observed in our study are not classically defined T_FH_ cells, nor are they the CXCR5+ central memory T cells that have been posited as a circulating counterpart to T_FH_ cells (38). While literature specific to CXCR5+ naïve CD4 T cells is scarce given that CXCR5 is primarily expressed by memory T populations, these cells may be precursors to T_FH_ or otherwise related to T cell/B cell interactions. Our observations in this celltype would complement existing literature demonstrating impaired T-cell dependent B cell function following Borrelial infection in mouse models (39, 40). The intriguing positive association of CXCR5+ naïve CD4 T cells with musculoskeletal symptoms in our PTLD cohort further links these cells to disease manifestations. Likely, such a link would involve indirect involvement of CXCR5+ naïve CD4 T cells and more proximally relate to B cell dysregulation or irregular lymph node dynamics which cannot be observed in peripheral blood, yet have demonstrable importance during acute infection (41).

Our observation of increased CD57+ CD4+ T_EMRA_ cells may support the presence of an inflammatory milieu, having been previously associated with cytotoxic function and expression of KLRG1 and CD154 (42–44). Included among theories of PTLD pathogenesis are the (non-exclusive) possibilities of persistent borrelial remnants or an autoimmune disorder initiated by events during acute infection (35). Both of these mechanisms could result in chronic inflammation, be consistent with elevated prevalence of cytotoxic cell types, and may contribute to fatigue and myalgias emblematic of PTLD.

Similarly to subpopulations of CD4+ helper T cells, CD8+ T cells have been characterized to have multiple effector phenotypes with distinct cytokine profiles (45, 46). We identified an increase in a Th1-like CD8+ population (CXCR3+ CCR4- CCR6-), with a corresponding decrease in Th1/17-like CD8+ cells (CXCR3-CCR4+CCR6+). We are cautious in applying Tc1 or Tc1/17 nomenclature, as functional cytokine secretion of these cells has not been established. However, it is possible these Th1-like CD8+ T cells are Tc1 cells, which secrete inflammatory cytokines including IFNγ and TNFα. Like CD57+ CD4+ T_EMRA_ cells discussed above, an increased presence of these cells would be consistent with a chronic inflammatory state similar to disease mechanisms observed in autoimmune conditions (47, 48). Under similar assumptions, if our observed CXCR3-CCR4+CCR6+ cells are Tc1/17 cells, their absence may suggest deficiencies in IL-17 production and dysregulated B cell responses consistent with CXCR5 expression as discussed previously. Although we did not observe differences in circulating IFNγ, TNFα, or IL-17, which might support the functional relevance of these observations, secretion of these cytokines is likely paracrine rather than systemic. IL-23, another relevant cytokine in T_fh_ biology which has been associated with Lyme infection, was not measured (9).

Previous symptom profiling in PTLD, along with the factor analysis reported here, demonstrate sex-specific differences in symptomatology. However, a comparison of immune features either (i) between healthy controls and PTLD participants or (ii) across symptom subgroups did not yield substantially different results when performed on either sex alone compared to the combined cohort. Our sole sex-specific observation is the female-specific increase in CD8 T_CM_ cells within patient subgroup 3. This subgroup was characterized by high fatigue/cognitive factor scores and a high incidence of flu-like illness upon initial infection. Increased T_CM_ cells may suggest a greater capacity for durable, proliferative responses following an inflammatory insult (49). Why this observation is restricted to the females of patient subgroup 3, but not males, is unclear and requires future study.

Summarily, the cell types most notably different between healthy controls and our PTLD cohort are suggestive of chronic inflammation and irregularities in T-cell help. We speculate that disease mechanisms related to our findings would be similar to those reported in autoimmunity and other post-acute infection syndromes (8, 24, 35, 50). Importantly, our cohort study does not include participants with Lyme disease who returned to health following antibiotic treatment of acute infection. This group would be important to distinguish pathologic features of PTLD from non-pathologic, long-term changes which may occur in all individuals following Lyme infection. However, if our observations are specific to PTLD, measurement of the aforementioned cell types could be diagnostically useful for this disease, which remains a strictly clinical diagnosis.

This study has several limitations given that our findings are observational, and in a retrospectively recruited, cross-sectional PTLD cohort. As mentioned, participant enrollment occurred long after acute infection in this cross-sectional study. Furthermore, while all patients met specified criteria for prior Lyme disease, not all patients had identical initial presentations, including not requiring an EM rash but meeting CDC criteria for Lyme disease based on symptoms and confirmatory serology (7, 25). Second, we note that the small number of healthy controls in this study reduces our power to resolve differences between healthy controls and PTLD, especially when split for sex-specific comparisons. An additional limitation is the collection of flow cytometry data on fresh blood over a multi-year period. Although freshly analyzed PBMCs have advantages over frozen samples, day-to-day assay variation may increase noise in the data. Lastly, while we analyzed many immunologic parameters, this study is far from exhaustive. T cell phenotypes were the most thoroughly explored in our flow cytometry panel, with a limited exploration of B cell, innate immune cell, and monocyte phenotypes.

For the *in silico* component of this work, it is possible that changes in our approach to clustering, or our data processing pipeline more broadly, might reveal additional insights relating symptom subgroups to immune alterations in PTLD including sex-based heterogeneity. While our choice of six PTLD symptom subgroups was guided by several statistical measures and clinically relevant observations, choosing the best cluster resolution in any dataset is a challenging problem dependent on the data, clustering algorithm used, and intended downstream applications (51). Further validation of our findings will be important in longitudinal Lyme disease cohorts and mechanistic studies employing high-dimensional methods to interrogate a broader range of cell types, and to more deeply explore the functional consequences of the differences we identified.

Collectively, we identified objective immune perturbations which both distinguish PTLD from healthy controls and associate with specific PTLD symptoms. Though observational in nature, our findings are consistent with literature supporting dysregulated humoral responses following borrelial infection, and an inflammatory/exhausted T cell profile similar to those observed in other chronic or post-acute infection syndromes (22, 35, 48). With further investigation and a comparison to return to health cohorts, assaying the immune populations identified in this manuscript may have diagnostic utility. In addition, our work lays the foundation for future mechanistic studies. Our strategy of factor analysis and patient subgroup profiling, followed by examining potential associations with immune features, provides a useful framework for future studies of post-infection syndrome cohorts which are so often heterogenous. As methodological advances in high-dimensional immunophenotyping and analytics continue, abnormalities underlying PTLD and its heterogenous manifestations will come more clearly into view.

## Data Availability

The raw data supporting the conclusions of this article will be made available by the authors, without undue reservation.
