# Supplementary material for: Aberrant T-cell phenotypes in a cohort of patients with post-treatment Lyme disease
Source: Front Immunol. 2025 Jul 9;16:1607619. doi: 10.3389/fimmu.2025.1607619 (PMC12283721; doi:10.3389/fimmu.2025.1607619)
Supplement: Supplementary file 9 [file SupplementaryFile1.pdf]

Supplementary Material

"Aberrant T-cell Phenotypes in a Cohort of Patients with Post-Treatment Lyme Disease," by Girgis et al. *Frontiers in Immunology* 2025

Figure S1	Subject cohorts and data overlap
Figure S2	Cytokine profile of k-means clusters.
Figure S3.	Representative gating strategy used to identify immune cell subsets
Figure S4.	Selected flow parameters by sex.
Table S1.	Soluble immune mediators measured using the Luminex platform
Table S2.	Markers used for flow cytometry panel.
Table S3.	Summary of elastic net regressions used to predict PLQS factor scores based on immunophenotyping data

Additional Files

Table 1.xlsx	PTLD and healthy control cohorts demographic summary
Table 2.xlsx	PLQS patient subgroup demographic summary
Table 3.xlsx	Cytokine/chemokine data summary
Table 4.xlsx	Cytokine/chemokine significance testing results
Table 5.xlsx	Cytokine/chemokine correlations with PLQS symptom scores
Table 6.xlsx	Flow cytometry data summary
Table 7.xlsx	Flow cytometry significance testing results
Table 8.xlsx	Flow cytometry correlations with PLQS symptom scores
Data Sheet 1.pdf	Flow cytometry gating strategy schematic
Data Sheet 2.pdf	Unabridged flow cytometry gating example

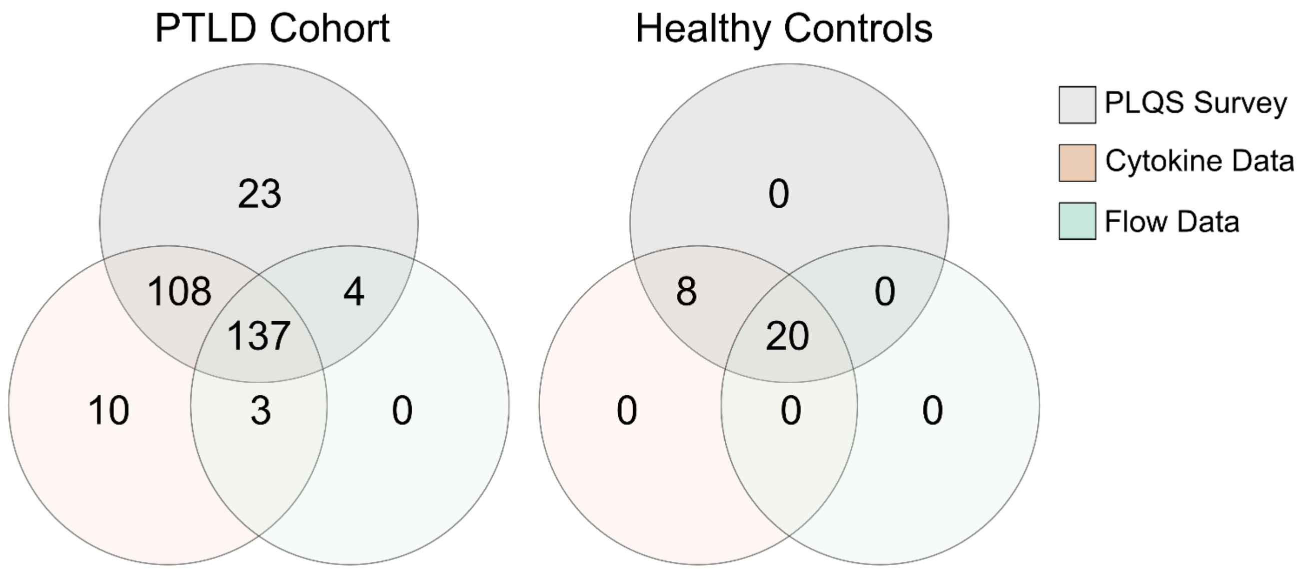

Figure S1. Subject cohorts and data overlap. Values within each section of the Venn diagram are the number of subjects for whom each data modality was present. 272 PTLD subjects completed the PLQS survey, 258 had sera submitted for cytokine analysis, and 144 were assayed via flow cytometry. Variable overlaps exist between cohorts; where possible, the maximum number of individuals were analyzed (i.e., 258 PTLD patents were used in significance testing of cytokine data, despite not all of subjects having been assayed using flow cytometry).

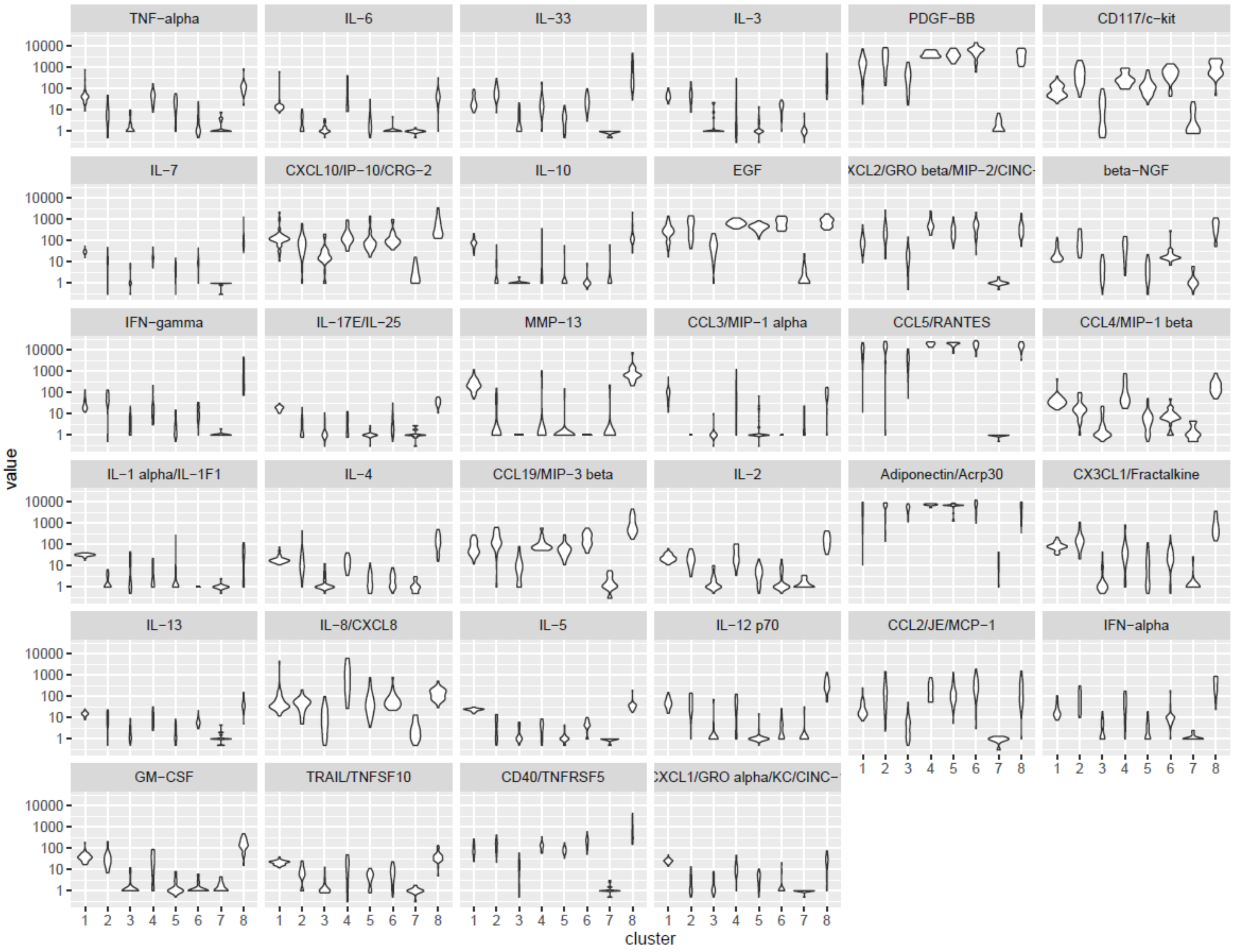

Figure S2. Cytokine profile of k-means clusters. Multivariate analysis was performed on cytokine data to generate 8 clusters of subjects with distinct cytokine expression profiles. These clusters did not separate PTLD subjects from healthy controls, or separate PTLD individuals by patient subgroup (Figure 2A).

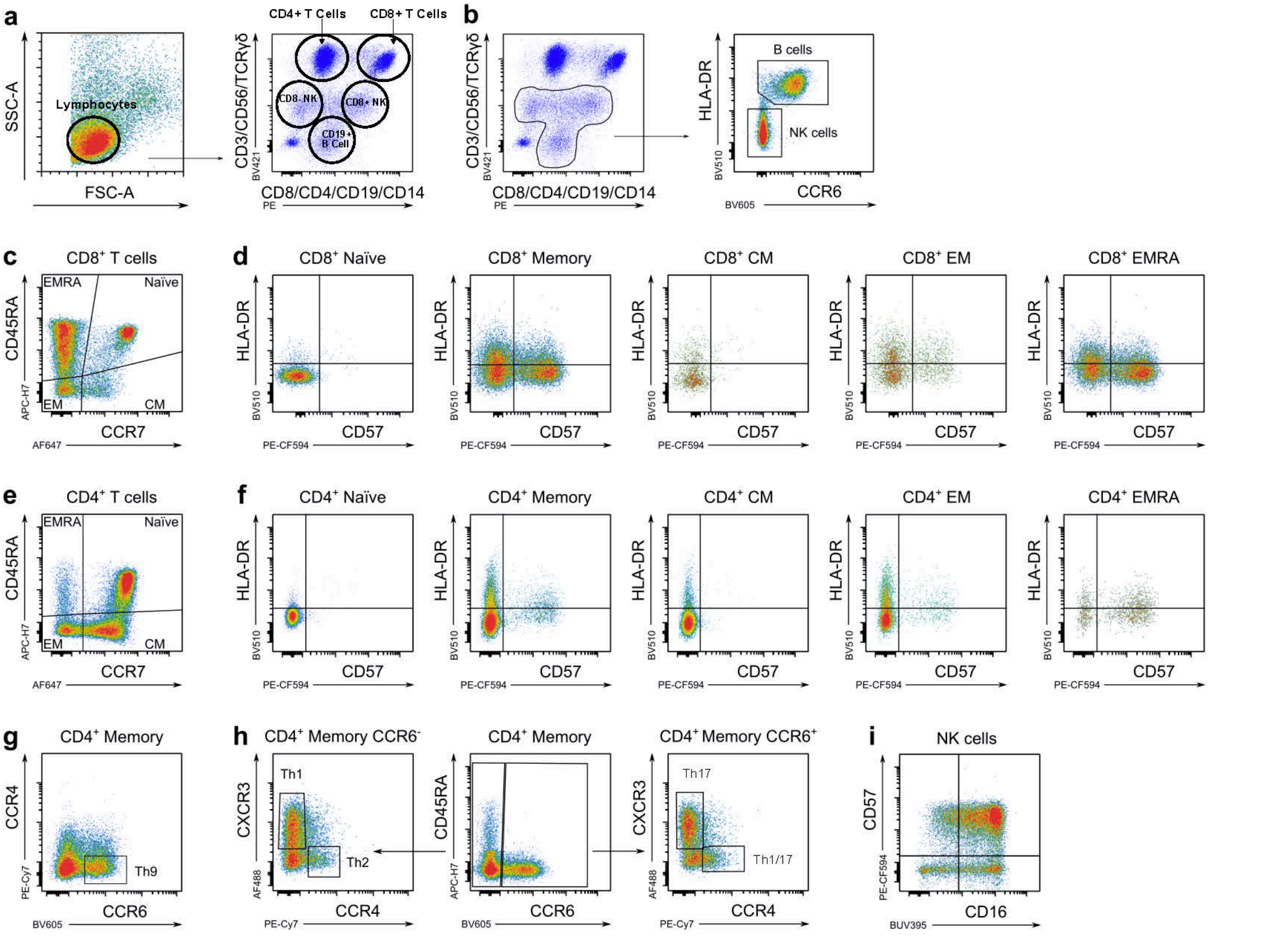

Figure S3. Representative gating strategy used to identify immune cell subsets. **(A)** Viable mononuclear cells were pre-gated to eliminate doublets and then lymphocytes (left panel) gated for expression of markers that identify key lymphocyte subsets as described in detail in reference 29. **(B)** B cells and NK cells can be further distinguished by differences in expression of CCR6 and HLA-DR. **(C)** CD8+ and **(E)** CD4+ T cells were further identified as naïve and memory subsets using expression of CD45RA and CCR7. Expression of the markers CD57 and HLA-DR were confined to memory CD8+ **(D)** and CD4+ T Cell subsets **(F)**. Note Memory T cells = total memory T cells (CM+EM+EMRA). Panels **(G)** and **(H)** display the strategy to identify effecter T cell subsets within total memory CD4+ T cells utilizing differential expression of CXCR3, CCR4 and CCR6. An identical strategy was utilized for CD8+ memory T cells (not shown). **(I)** NK cells subsets can be further identified using CD57 and CD16 expression.

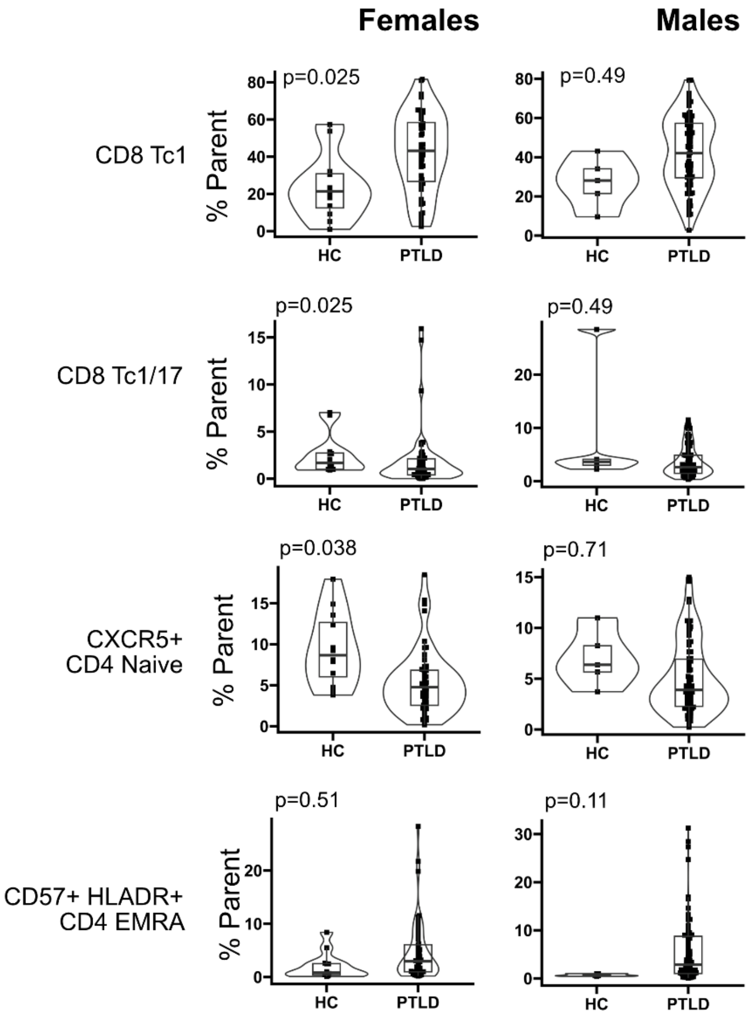

Figure S4. Selected flow parameters by sex. Parameters which were significantly different between PTLD and healthy controls in combined-sex testing had the same direction of change in each sex when tested separately.

Table S1. Soluble immune mediators measured using the Luminex platform. Thirty-four analytes were measured in the sera of 258 PTLD patients and 28 healthy controls.

Analyte	
Adiponectin	IL-1 alpha
beta-NGF	IL-10
CCL19	IL-12 p70
CCL2	IL-13
CCL3	IL-17E/IL-25
CCL4	IL-2
CCL5	IL-3
CD117	IL-33
CD40	IL-4
CX3CL1	IL-5
CXCL1	IL-6
CXCL10	IL-7
CXCL2	IL-8
EGF	MMP-13
GM-CSF	PDGF-BB
IFN-alpha	TNF-alpha
IFN-gamma	TRAIL/TNFSF10

Table S2. Markers used for flow cytometry panel. The panel was applied to freshly isolated PBMCs from 144 PTLD patients and 20 healthy controls. 16 monoclonal antibodies, viability dye, and forward/side scatter were used to generate 119 gated cell populations.

Marker	Fluorophore	Cat No.	Clone
CD3	BV421	BD 562426	UCHT1
CD56	BV421	BD 562751	NCAM16.2
TCRgd	BV421	Biolegend 331217	B1
CD4	PE	BD 555347	RPA-T4
CD8	PE	BD 555367	RPA-T8
CD14	PE	BD 555398	M5E2
CD19	PE	BD 555413	HIB19
CD16	BUV395	BD 563784	3G8
CXCR5	PerCP-Cy5.5	BD 562781	RF8B2
CD57	PE Dazzle594	Biolegend 359619	HNK-1
HLADR	BV510	BD 563083	G46-6
CCR6	BV605	Biolegend 353420	G034E3
CXCR3	AF488	BD 558047	1C6/CXCR3
CCR4	PE-Cy7	BD 557864	1G1
CCR7	AF647	Biolegend 353218	G043H7
CD45RA	APC-H7	BD 560674	HI100
Viability	Live Dead Fixable Blue	Thermo Fisher L34962	N/A

Table S3. Summary of elastic net regressions used to predict PLQS factor scores based on immunophenotyping data. One hundred models were attempted for each PLQS factor score. Models were unable to predict PLQS scores with any accuracy, and in many iterations failed to generate non-zero coefficients.

PLQS Factor	Median Spearman Correlation	Proportion Failed Models
**Neurologic**	0.0423	45/100
**Fatigue/Cognitive**	0.0001	39/100
**Infection-type**	-0.0966	61/100
**Ocular Disequilibrium**	0.075	54/100
**Musculoskeletal Pain**	0.1183	48/100
**Mood-related**	0.0166	49/100
