# Supplementary material for: Aberrant T-cell phenotypes in a cohort of patients with post-treatment Lyme disease
Source: Front Immunol. 2025 Jul 9;16:1607619. doi: 10.3389/fimmu.2025.1607619 (PMC12283721; doi:10.3389/fimmu.2025.1607619)
Supplement: Supplementary file 10 [file DataSheet1.pdf]

First Layer Subsets

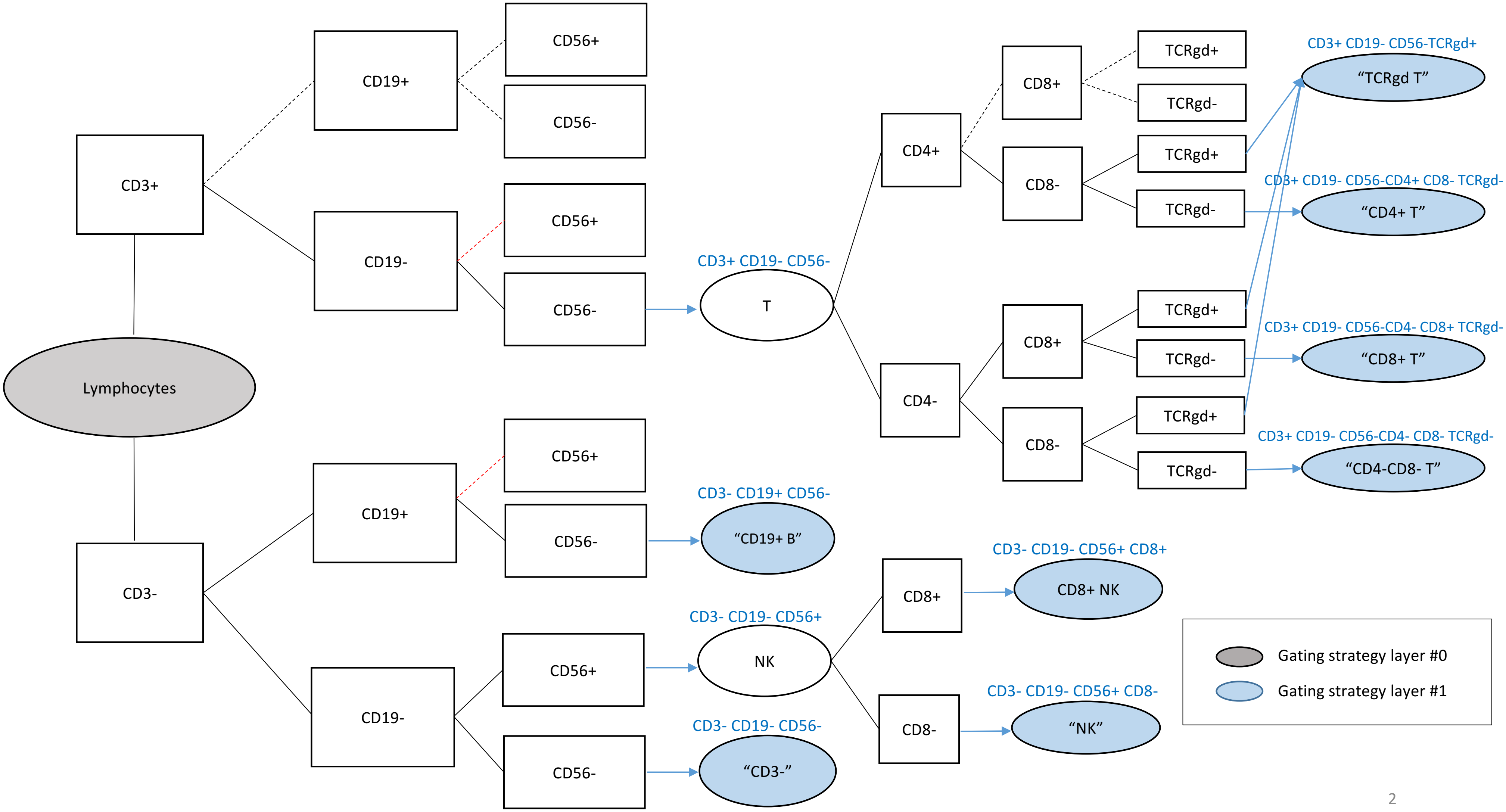
Lymphocytes
CD3+
CD19+
CD56+
CD56-
CD19-
CD56+
CD56-
CD3+ CD19- CD56-
T
CD4+
CD8+
TCRgd+
TCRgd-
CD8-
TCRgd+
TCRgd-
CD4-
CD8+
TCRgd+
TCRgd-
CD8-
TCRgd+
TCRgd-
CD3+ CD19- CD56-TCRgd+
"TCRgd T"
CD3+ CD19- CD56-CD4+ CD8- TCRgd-
"CD4+ T"
CD3+ CD19- CD56-CD4- CD8+ TCRgd-
"CD8+ T"
CD3+ CD19- CD56-CD4- CD8- TCRgd-
"CD4-CD8- T"
CD3-
CD19+
CD56+
CD56-
CD3- CD19+ CD56-
"CD19+ B"
CD19-
CD56+
CD56-
CD3- CD19- CD56+
NK
CD3- CD19- CD56-
"CD3-"
CD8+
CD3- CD19- CD56+ CD8+
CD8+ NK
CD8-
CD3- CD19- CD56+ CD8-
"NK"
Gating strategy layer #0
Gating strategy layer #1

Second Layer Subsets

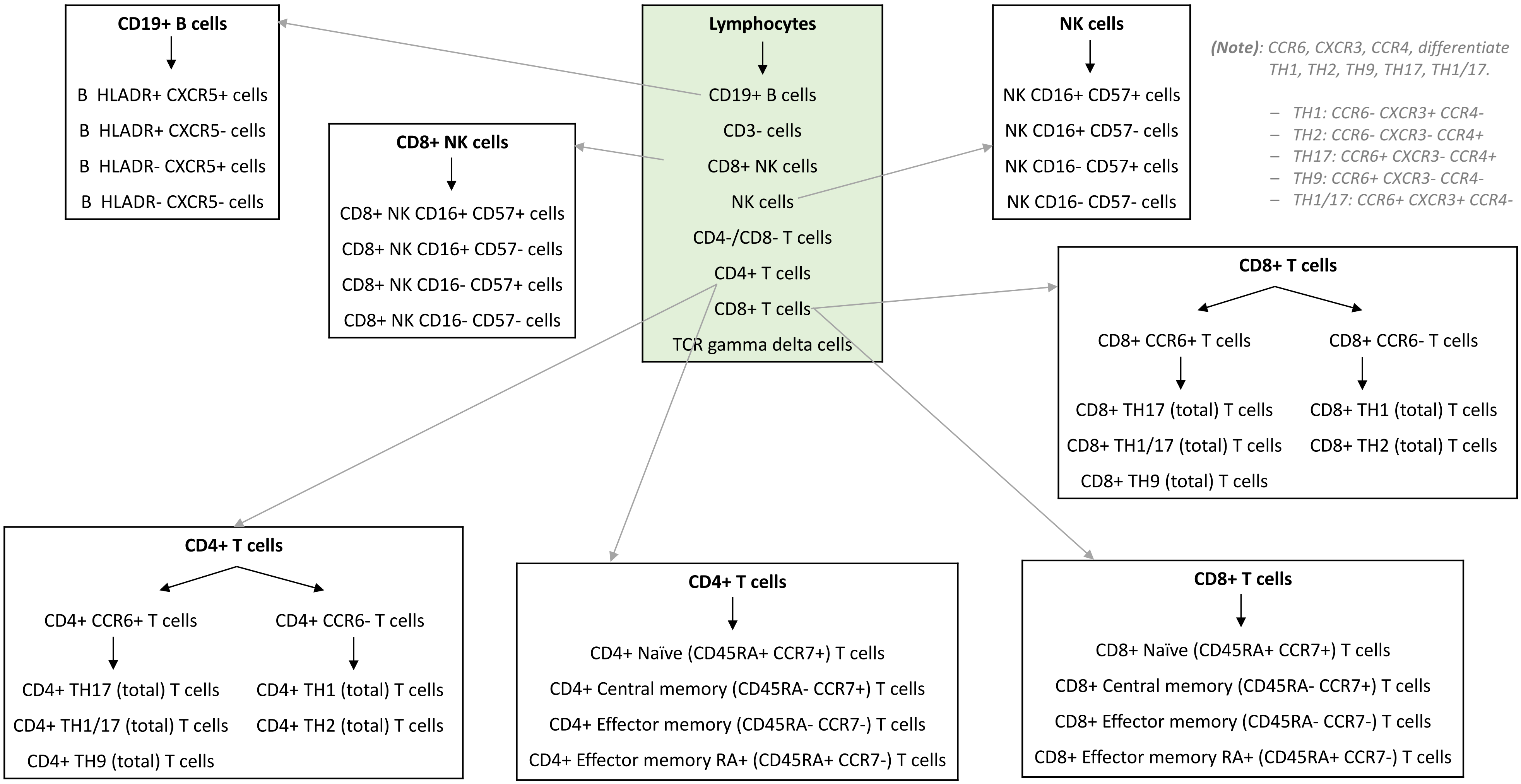
CD19+ B cells
B HLADR+ CXCR5+ cells
B HLADR+ CXCR5- cells
B HLADR- CXCR5+ cells
B HLADR- CXCR5- cells
Lymphocytes
CD19+ B cells
CD3- cells
CD8+ NK cells
NK cells
CD4-/CD8- T cells
CD4+ T cells
CD8+ T cells
TCR gamma delta cells
NK cells
NK CD16+ CD57+ cells
NK CD16+ CD57- cells
NK CD16- CD57+ cells
NK CD16- CD57- cells
(Note): CCR6, CXCR3, CCR4, differentiate TH1, TH2, TH9, TH17, TH1/17.
– TH1: CCR6- CXCR3+ CCR4-
– TH2: CCR6- CXCR3- CCR4+
– TH17: CCR6+ CXCR3- CCR4+
– TH9: CCR6+ CXCR3- CCR4-
– TH1/17: CCR6+ CXCR3+ CCR4-
CD8+ NK cells
CD8+ NK CD16+ CD57+ cells
CD8+ NK CD16+ CD57- cells
CD8+ NK CD16- CD57+ cells
CD8+ NK CD16- CD57- cells
CD8+ T cells
CD8+ CCR6+ T cells
CD8+ CCR6- T cells
CD8+ TH17 (total) T cells
CD8+ TH1/17 (total) T cells
CD8+ TH9 (total) T cells
CD8+ TH1 (total) T cells
CD8+ TH2 (total) T cells
CD4+ T cells
CD4+ CCR6+ T cells
CD4+ CCR6- T cells
CD4+ TH17 (total) T cells
CD4+ TH1/17 (total) T cells
CD4+ TH9 (total) T cells
CD4+ TH1 (total) T cells
CD4+ TH2 (total) T cells
CD4+ T cells
CD4+ Naïve (CD45RA+ CCR7+) T cells
CD4+ Central memory (CD45RA- CCR7+) T cells
CD4+ Effector memory (CD45RA- CCR7-) T cells
CD4+ Effector memory RA+ (CD45RA+ CCR7-) T cells
CD8+ T cells
CD8+ Naïve (CD45RA+ CCR7+) T cells
CD8+ Central memory (CD45RA- CCR7+) T cells
CD8+ Effector memory (CD45RA- CCR7-) T cells
CD8+ Effector memory RA+ (CD45RA+ CCR7-) T cells

Third Layer Subsets (CD4+ T Cells)

CD4+ Naïve T cells

CD4+ CCR6+ Naïve T cells
- CD4+ TH17 Naïve T cells
- CD4+ TH1/17 Naïve T cells
- CD4+ TH9 Naïve T cells

CD4+ CCR6- Naïve T cells
- CD4+ TH1 Naïve T cells
- CD4+ TH2 Naïve T cells

CD4+ Central Memory (CM) T cells

CD4+ CCR6+ CM T cells
- CD4+ TH17 CM T cells
- CD4+ TH1/17 CM T cells
- CD4+ TH9 CM T cells

CD4+ CCR6- CM T cells
- CD4+ TH1 CM T cells
- CD4+ TH2 CM T cells

CD4+ Effector Memory (EM) T cells

CD4+ CCR6+ EM T cells
- CD4+ TH17 EM T cells
- CD4+ TH1/17 EM T cells
- CD4+ TH9 EM T cells

CD4+ CCR6- EM T cells
- CD4+ TH1 EM T cells
- CD4+ TH2 EM T cells

CD4+ Naïve T cells

CD4+ HLADR+ CD57+ Naïve T cells

CD4+ HLADR+ CD57- Naïve T cells

CD4+ HLADR- CD57+ Naïve T cells

CD4+ HLADR- CD57- Naïve T cells

CD4+ Central Memory (CM) T cells

CD4+ HLADR+ CD57+ CM T cells

CD4+ HLADR+ CD57- CM T cells

CD4+ HLADR- CD57+ CM T cells

CD4+ HLADR- CD57- CM T cells

CD4+ Effector Memory (EM) T cells

CD4+ HLADR+ CD57+ EM T cells

CD4+ HLADR+ CD57- EM T cells

CD4+ HLADR- CD57+ EM T cells

CD4+ HLADR- CD57- EM T cells

CD4+ Naïve T cells

CD4+ CD45RA+ CXCR5+ Naïve T cells
CD4+ CD45RA+ CXCR5- Naïve T cells
~~CD4+ CD45RA- CXCR5+ Naïve T cells~~
~~CD4+ CD45RA- CXCR5- Naïve T cells~~

CD4+ Central Memory (CM) T cells

~~CD4+ CD45RA+ CXCR5+ CM T cells~~
~~CD4+ CD45RA+ CXCR5- CM T cells~~
CD4+ CD45RA- CXCR5+ CM T cells
CD4+ CD45RA- CXCR5- CM T cells

CD4+ Effector Memory (EM) T cells

~~CD4+ CD45RA+ CXCR5+ EM T cells~~
~~CD4+ CD45RA+ CXCR5- EM T cells~~
CD4+ CD45RA- CXCR5+ EM T cells
CD4+ CD45RA- CXCR5- EM T cells

(Note 1)*: 99.9% of the CD4+ Naïve T cells are CXCR5-*
(Note 2)*: CD4+ Naïve T cells should rarely express HLADR or CD57*
(Note 3)*: In the flow cytometry dataset, all percentages will be % out of direct parent node. For TH cells, we will also calculate % out of CD4+ memory T cells (grandparent)*
(Note 4)*: CD4+ CXCR5+ T cells are T_{TH} cells*
(Note 5)*: Naïve and EMRA cells are CD45RA+, and CM and EM cells are CD45RA-*

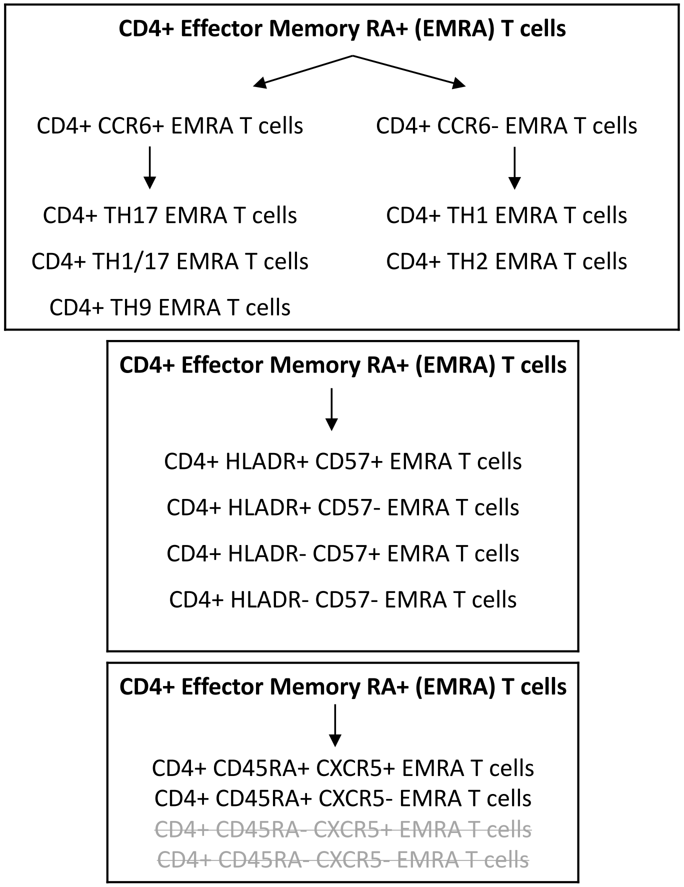
CD4+ Effector Memory RA+ (EMRA) T cells
CD4+ CCR6+ EMRA T cells
CD4+ CCR6- EMRA T cells
CD4+ TH17 EMRA T cells
CD4+ TH1/17 EMRA T cells
CD4+ TH9 EMRA T cells
CD4+ TH1 EMRA T cells
CD4+ TH2 EMRA T cells
CD4+ Effector Memory RA+ (EMRA) T cells
CD4+ HLADR+ CD57+ EMRA T cells
CD4+ HLADR+ CD57- EMRA T cells
CD4+ HLADR- CD57+ EMRA T cells
CD4+ HLADR- CD57- EMRA T cells
CD4+ Effector Memory RA+ (EMRA) T cells
CD4+ CD45RA+ CXCR5+ EMRA T cells
CD4+ CD45RA+ CXCR5- EMRA T cells
~~CD4+ CD45RA- CXCR5+ EMRA T cells~~
~~CD4+ CD45RA- CXCR5- EMRA T cells~~

Third Layer Subsets (CD8+ T Cells)

CD8+ Naïve T cells

CD8+ CCR6+ Naïve T cells
- CD8+ TH17 Naïve T cells
- CD8+ TH1/17 Naïve T cells
- CD8+ TH9 Naïve T cells

CD8+ CCR6- Naïve T cells
- CD8+ TH1 Naïve T cells
- CD8+ TH2 Naïve T cells

CD8+ Central Memory (CM) T cells

CD8+ CCR6+ CM T cells
- CD8+ TH17 CM T cells
- CD8+ TH1/17 CM T cells
- CD8+ TH9 CM T cells

CD8+ CCR6- CM T cells
- CD8+ TH1 CM T cells
- CD8+ TH2 CM T cells

CD8+ Effector Memory (EM) T cells

CD8+ CCR6+ EM T cells
- CD8+ TH17 EM T cells
- CD8+ TH1/17 EM T cells
- CD8+ TH9 EM T cells

CD8+ CCR6- EM T cells
- CD8+ TH1 EM T cells
- CD8+ TH2 EM T cells

CD8+ Naïve T cells

CD8+ HLADR+ CD57+ Naïve T cells
CD8+ HLADR+ CD57- Naïve T cells
CD8+ HLADR- CD57+ Naïve T cells
CD8+ HLADR- CD57- Naïve T cells

CD8+ Central Memory (CM) T cells

CD8+ HLADR+ CD57+ CM T cells
CD8+ HLADR+ CD57- CM T cells
CD8+ HLADR- CD57+ CM T cells
CD8+ HLADR- CD57- CM T cells

CD8+ Effector Memory (EM) T cells

CD8+ HLADR+ CD57+ EM T cells
CD8+ HLADR+ CD57- EM T cells
CD8+ HLADR- CD57+ EM T cells
CD8+ HLADR- CD57- EM T cells

CD8+ Naïve T cells

CD8+ CD45RA+ CXCR5+ Naïve T cells
CD8+ CD45RA+ CXCR5- Naïve T cells
~~CD8+ CD45RA+ CXCR5- Naïve T cells~~
~~CD8+ CD45RA+ CXCR5- Naïve T cells~~

CD8+ Central Memory (CM) T cells

~~CD8+ CD45RA+ CXCR5+ CM T cells~~
~~CD8+ CD45RA+ CXCR5- CM T cells~~
CD8+ CD45RA- CXCR5+ CM T cells
CD8+ CD45RA- CXCR5- CM T cells

CD8+ Effector Memory (EM) T cells

~~CD8+ CD45RA+ CXCR5+ EM T cells~~
~~CD8+ CD45RA+ CXCR5- EM T cells~~
CD8+ CD45RA- CXCR5+ EM T cells
CD8+ CD45RA- CXCR5- EM T cells

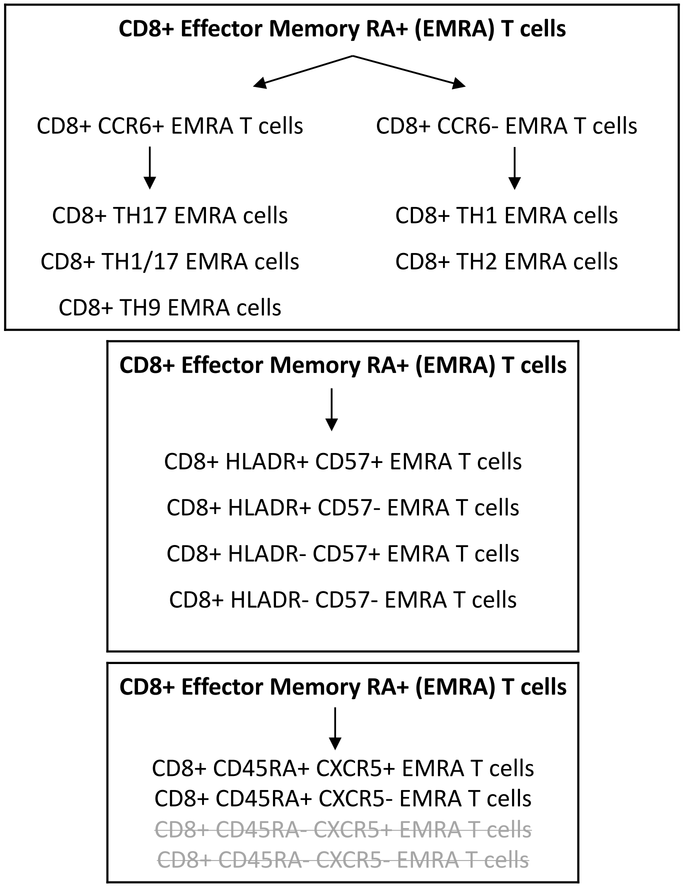
CD8+ Effector Memory RA+ (EMRA) T cells
CD8+ CCR6+ EMRA T cells
CD8+ CCR6- EMRA T cells
CD8+ TH17 EMRA cells
CD8+ TH1/17 EMRA cells
CD8+ TH9 EMRA cells
CD8+ TH1 EMRA cells
CD8+ TH2 EMRA cells
CD8+ Effector Memory RA+ (EMRA) T cells
CD8+ HLADR+ CD57+ EMRA T cells
CD8+ HLADR+ CD57- EMRA T cells
CD8+ HLADR- CD57+ EMRA T cells
CD8+ HLADR- CD57- EMRA T cells
CD8+ Effector Memory RA+ (EMRA) T cells
CD8+ CD45RA+ CXCR5+ EMRA T cells
CD8+ CD45RA+ CXCR5- EMRA T cells
~~CD8+ CD45RA- CXCR5+ EMRA T cells~~
~~CD8+ CD45RA- CXCR5- EMRA T cells~~
