# Supplementary material for: Aberrant T-cell phenotypes in a cohort of patients with post-treatment Lyme disease
Source: Front Immunol. 2025 Jul 9;16:1607619. doi: 10.3389/fimmu.2025.1607619 (PMC12283721; doi:10.3389/fimmu.2025.1607619)
Supplement: Supplementary file 11 [file DataSheet2.pdf]

Lymphocytes

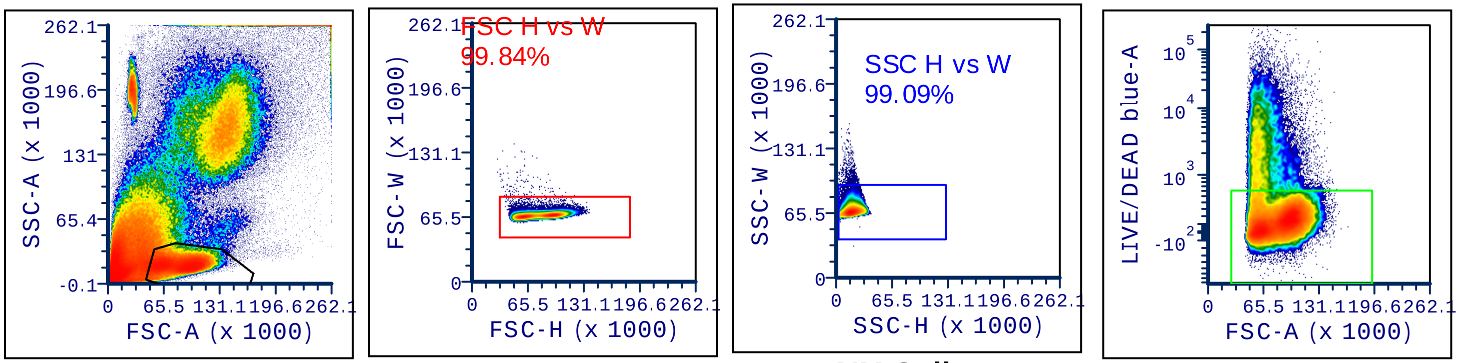

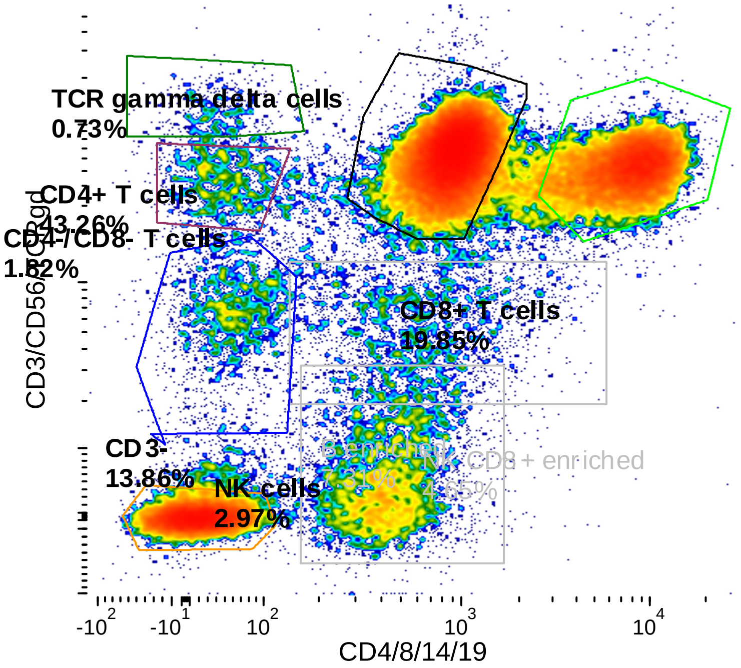

Population	% of Gated Cells	# of Events
TCR gamma delta	0.73	468
CD4+ T cells	43.26	27907
CD8+ T cells	19.85	12804
CD4-/CD8- T cells	1.82	1172
NK cells	2.97	1918
CD8+ NK cells	2.32	1498
CD3- cells	13.86	8945
CD19+ B cells	6.96	4491

NK Cells

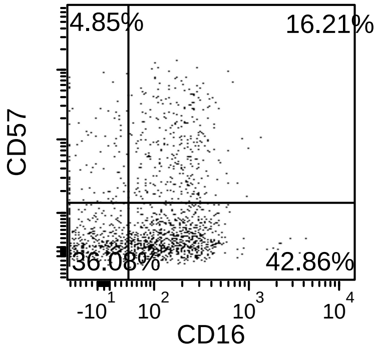

Population	% of Gated Cells	# of Events
CD16-/57-	36.08%	692
CD16+/57-	42.86%	822
CD16+/57+	16.21%	311
CD16-/57+	4.85%	93

CD8+ NK Cells

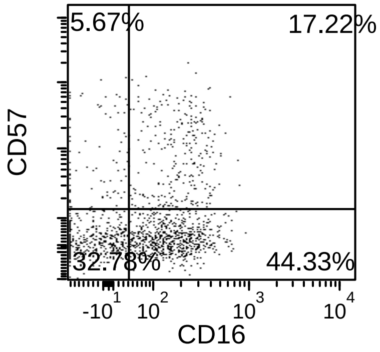

Population	% of Gated Cells	# of Events
CD16-/57-	32.78%	491
CD16+/57-	44.33%	664
CD16+/57+	17.22%	258
CD16-/57+	5.67%	85

B Cells

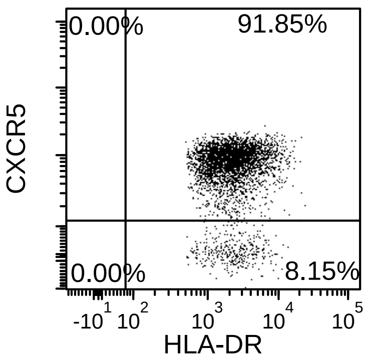

Population	% of Gated Cells	# of Events
DR-/CXCR5-	0.00%	0
DR+/CXCR5-	8.15%	366
DR+/CXCR5+	91.85%	4125
DR-/CXCR5+	0.00%	0

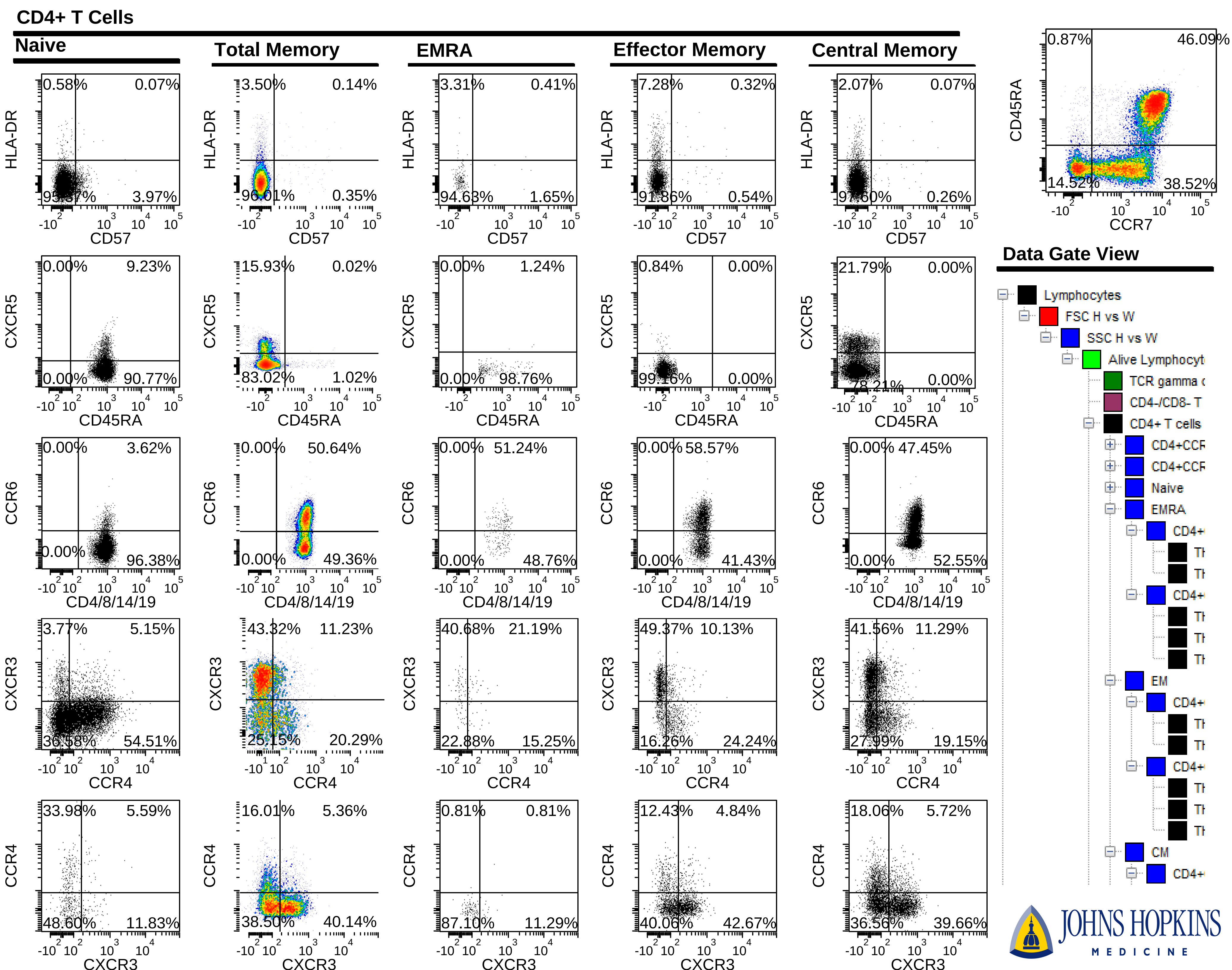

JOHNS HOPKINS
MEDICINE

CD4+ T Cells

	Naive		Total Memory		EMRA		Effector Memory		Central Memory	
	% Gated	# Events	% Gated	# Events	% Gated	# Events	% Gated	# Events	% Gated	# Events
CD57-DR-	95.37%	12267	96.01%	14445	94.63%	229	91.86%	3723	97.60%	10492
CD57+DR-	3.97%	511	0.35%	53	1.65%	4	0.54%	22	0.26%	28
CD57+DR+	0.07%	9	0.14%	21	0.41%	1	0.32%	13	0.07%	7
CD57-DR+	0.58%	75	3.50%	526	3.31%	8	7.28%	295	2.07%	223
RA-CXCR5-	0.00%	0	83.02%	12491	0.00%	0	99.16%	4019	78.21%	8408
RA+CXCR5-	90.77%	11675	1.02%	154	98.76%	239	0.00%	0	0.00%	0
RA+CXCR5+	9.23%	1187	0.02%	3	1.24%	3	0.00%	0	0.00%	0
RA-CXCR5+	0.00%	0	15.93%	2397	0.00%	0	0.84%	34	21.79%	2342
CD4+CCR6-	96.38%	12397	49.36%	7426	48.76%	118	41.43%	1679	52.55%	5649
CD4+CCR6+	3.62%	465	50.64%	7619	51.24%	124	58.57%	2374	47.45%	5101
TH1	3.77%	467	43.32%	3217	40.68%	48	49.37%	829	41.56%	2348
TH2	54.51%	6757	20.29%	1507	15.25%	18	24.24%	407	19.15%	1082
TH17	33.98%	158	16.01%	1220	0.81%	1	12.43%	295	18.06%	921
TH9	48.60%	226	38.50%	2933	87.10%	273	40.06%	951	36.56%	1865
TH1/17	11.83%	55	40.14%	3058	11.29%	14	42.67%	1013	39.66%	2023

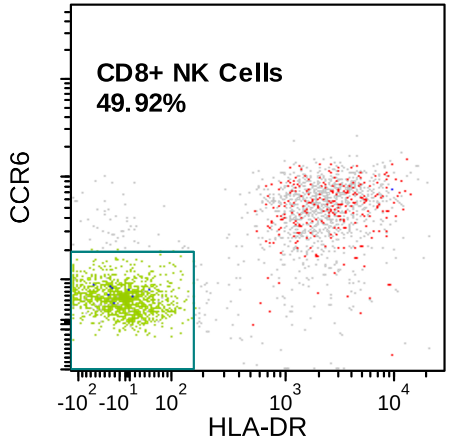

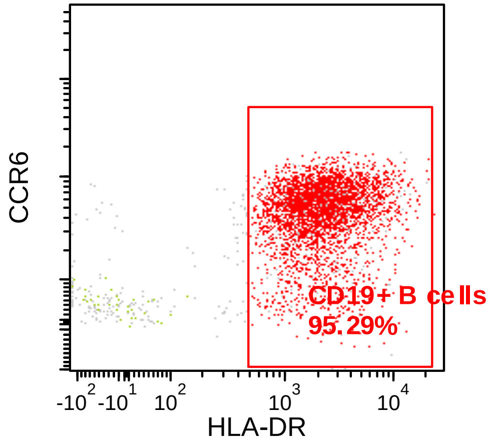

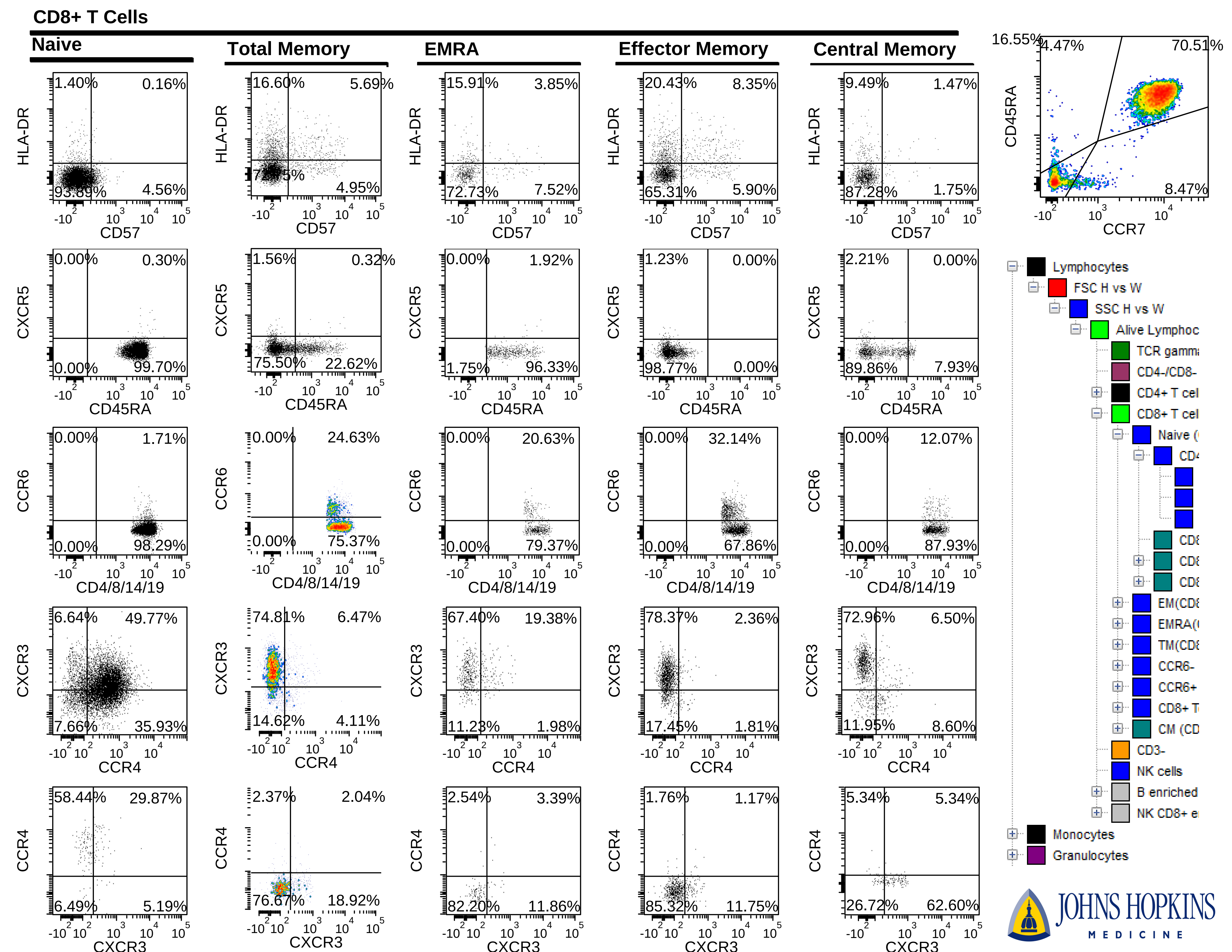
CD8+ T Cells
Naive
Total Memory
EMRA
Effector Memory
Central Memory
HLA-DR
CD57
1.40% 0.16% 93.89% 4.56%
16.60% 5.69% 4.95%
15.91% 3.85% 72.73% 7.52%
20.43% 8.35% 65.31% 5.90%
9.49% 1.47% 87.28% 1.75%
CXCR5
CD45RA
0.00% 0.30% 0.00% 99.70%
1.56% 0.32% 75.50% 22.62%
0.00% 1.92% 1.75% 96.33%
1.23% 0.00% 98.77% 0.00%
2.21% 0.00% 89.86% 7.93%
CCR6
CD4/8/14/19
0.00% 1.71% 0.00% 98.29%
0.00% 24.63% 0.00% 75.37%
0.00% 20.63% 0.00% 79.37%
0.00% 32.14% 0.00% 67.86%
0.00% 12.07% 0.00% 87.93%
CXCR3
CCR4
6.64% 49.77% 7.66% 35.93%
74.81% 6.47% 14.62% 4.11%
67.40% 19.38% 11.23% 1.98%
78.37% 2.36% 17.45% 1.81%
72.96% 6.50% 11.95% 8.60%
CCR4
CXCR3
58.44% 29.87% 6.49% 5.19%
2.37% 2.04% 76.67% 18.92%
2.54% 3.39% 82.20% 11.86%
1.76% 1.17% 85.32% 11.75%
5.34% 5.34% 26.72% 62.60%
CD45RA
CCR7
16.55% 4.47% 70.51% 8.47%
Lymphocytes
FSC H vs W
SSC H vs W
Alive Lymphoc
TCR gamm
CD4-/CD8-
CD4+ T cel
CD8+ T cel
Naive (
EM(CD8
EMRA(
TM(CD8
CCR6-
CCR6+
CD8+ T
CM (CD
CD3-
NK cells
B enriched
NK CD8+ e
Monocytes
Granulocytes
JOHNS HOPKINS
MEDICINE

CD8+ T Cells

	Naive		Total Memory		EMRA		Effector Memory		Central Memory	
	% Gated	# Events	% Gated	# Events	% Gated	# Events	% Gated	# Events	% Gated	# Events
CD57-DR-	93.89%	8476	72.75%	2747	72.73%	416	65.31%	1384	87.28%	947
CD57+DR-	4.56%	412	4.95%	187	7.52%	43	5.90%	125	1.75%	19
CD57+DR+	0.16%	14	5.69%	215	3.85%	22	8.35%	177	1.47%	16
CD57-DR+	1.40%	126	16.60%	627	15.91%	91	20.43%	433	9.49%	103
RA-CXCR5-	0.00%	0	75.50%	2851	1.75%	10	98.77%	2093	89.86%	975
RA+CXCR5-	99.70%	9001	22.62%	854	96.33%	551	0.00%	0	7.93%	86
RA+CXCR5+	0.30%	27	0.32%	12	1.92%	11	0.00%	0	0.00%	0
RA-CXCR5+	0.00%	0	1.56%	59	0.00%	0	1.23%	26	2.21%	24
CD4+CCR6-	98.29%	8874	75.37%	2846	79.37%	454	67.86%	1438	87.93%	954
CD4+CCR6+	1.71%	154	24.63%	930	20.63%	118	32.14%	681	12.07%	131
TH1	6.64%	589	74.81%	2129	67.40%	306	78.37%	1127	72.96	696
TH2	35.93%	3188	4.11%	117	1.98%	9	1.81%	26	8.60	82
TH17	58.44%	90	2.37%	22	2.54%	3	1.76%	12	5.34	7
TH9	6.49%	10	76.67%	713	82.20%	97	85.32%	581	26.72	35
TH1/17	5.19%	8	18.92%	176	11.86%	14	11.75%	80	62.60	82

CD4+ T Cells (Total)

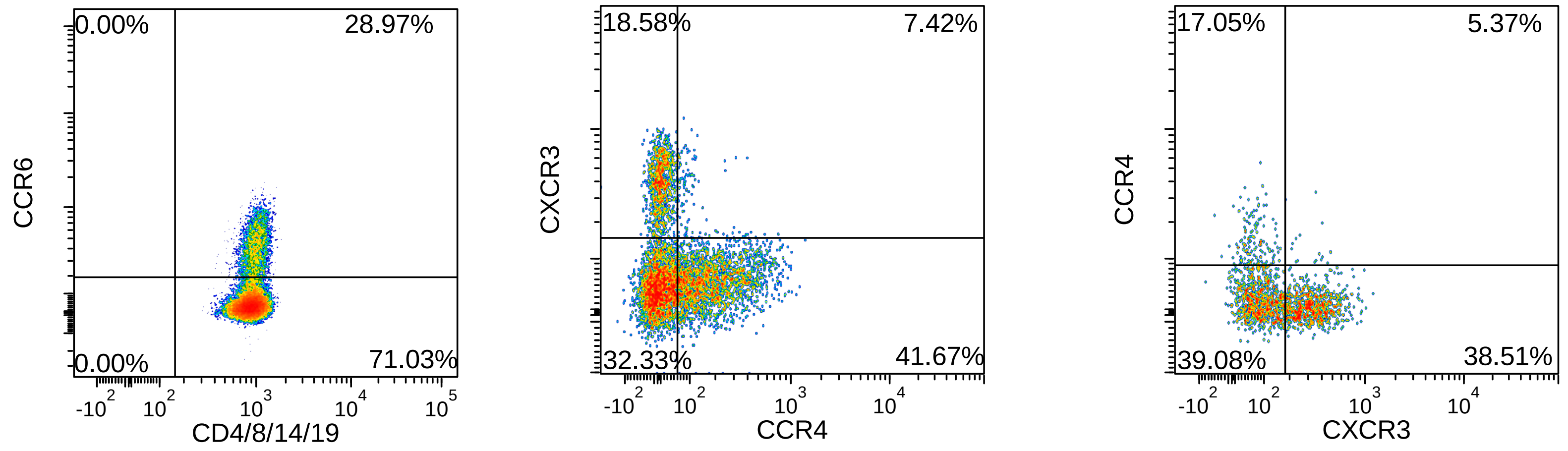

CD8+ T Cells (Total)

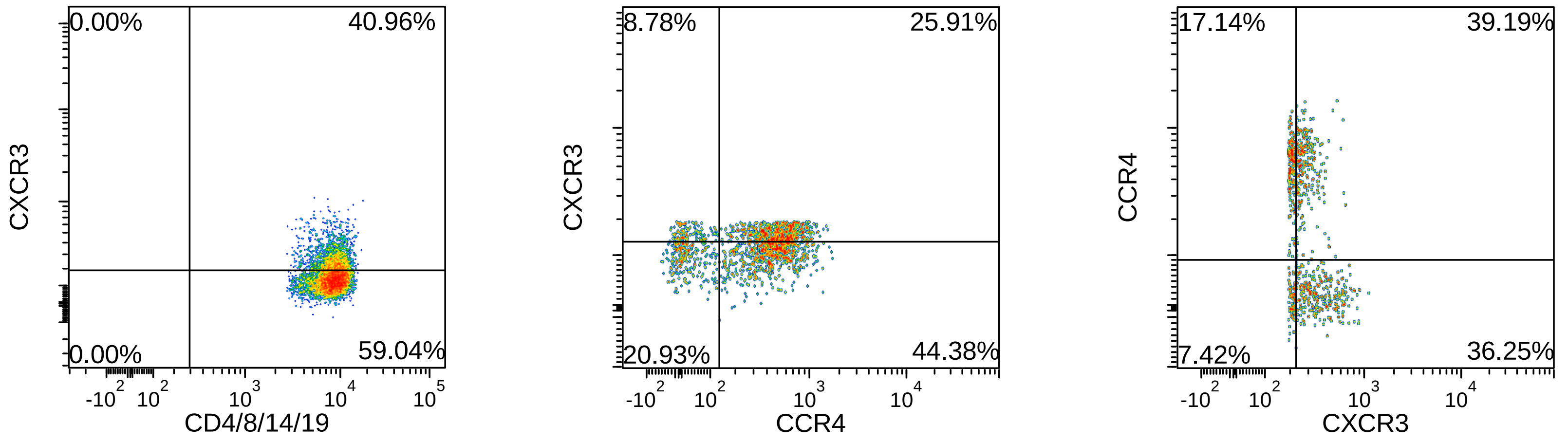

Data Gate View

- Lymphocytes
  - FSC H vs W
    - SSC H vs W
      - Alive Lymphocytes
        - TCR gamma delta c
        - CD4-/CD8- T cells
        - CD4+ T cells
          - CD4+CCR6-
            - TH1
            - TH2
          - CD4+CCR6+
            - TH1/17
            - TH17
            - TH9
          - Naive
          - EMRA
          - EM
          - CM
          - CD4+ Total Mer
            - CD4+CCR6
            - CD4+CCR6
              - TH17 (
              - TH9 (T
              - TH1/17
        - CD8+ T cells
          - Naive (CD8T)
            - CD4+CCR6
            - CD8+CCR6
            - CD8+ CCR6

JOHNS HOPKINS
MEDICINE

Monocytes

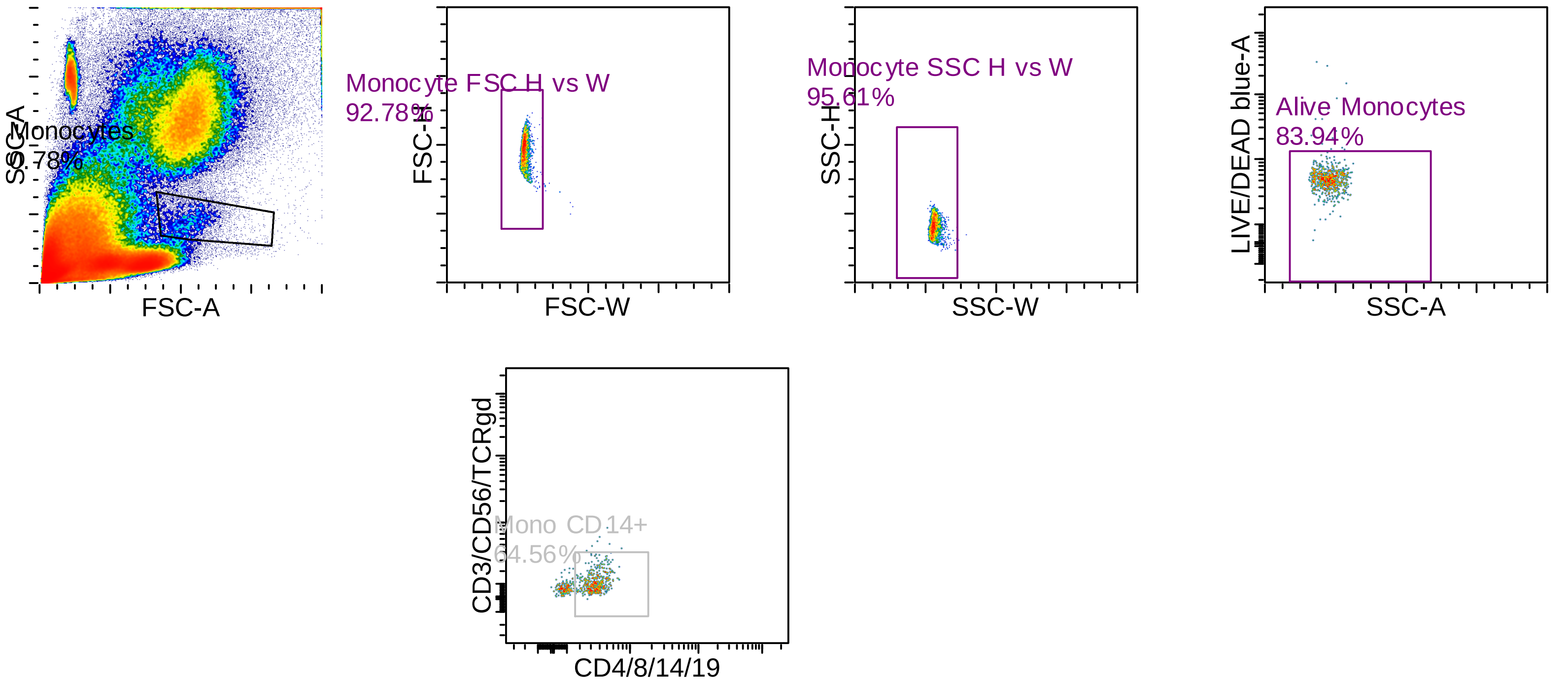

Granulocytes

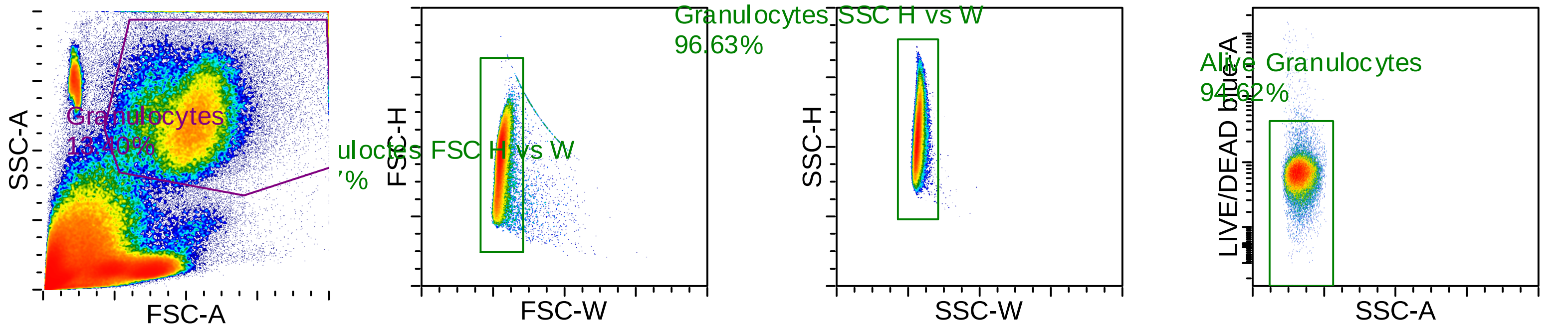

Data Gate View

- Lymphocytes
  - FSC H vs W
    - SSC H vs W
      - Alive Lymphocytes
        - TCR gamma delta c
        - CD4-/CD8- T cells
        - CD4+ T cells
        - CD8+ T cells
        - CD3-
        - NK cells
        - B enriched
        - NK CD8+ enriched
- Monocytes
  - Monocyte FSC H vs W
    - Monocyte SSC H vs W
      - Alive Monocytes
        - Mono CD14+
- Granulocytes
  - Granuloctes FSC H vs W
    - Granulocytes SSC H vs W
      - Alive Granulocytes
- beads

Calculated Concentrations

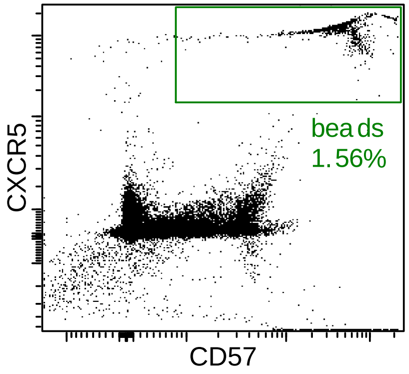

Concentration of Samples(cells/uL) = A/B*C/D
where A= number of cell events
B=number of bead events
C=assigned bead count/50uL
D=volume of sample

Concentration of sample
=16329.88 cells/uL
